# Leveraging the T2T assembly to resolve rare and pathogenic inversions in reference genome gaps

**DOI:** 10.1101/gr.279346.124

**Published:** 2024-11

**Authors:** Kristine Bilgrav Saether, Jesper Eisfeldt, Jesse D. Bengtsson, Ming Yin Lun, Christopher M. Grochowski, Medhat Mahmoud, Hsiao-Tuan Chao, Jill A. Rosenfeld, Pengfei Liu, Marlene Ek, Jakob Schuy, Adam Ameur, Hongzheng Dai, Undiagnosed Diseases Network, James Paul Hwang, Fritz J. Sedlazeck, Weimin Bi, Ronit Marom, Josephine Wincent, Ann Nordgren, Claudia M.B. Carvalho, Anna Lindstrand

**Affiliations:** 1 Department of Molecular Medicine and Surgery, Karolinska Institute, 171 76 Stockholm, Sweden;; 2 Science for Life Laboratory, Karolinska Insitutet, 171 65 Solna, Sweden;; 3 Department of Clinical Genetics and Genomics, Karolinska University Hospital, 171 76 Stockholm, Sweden;; 4 Pacific Northwest Research Institute, Seattle, Washington 98122, USA;; 5 Department of Molecular and Human Genetics, Baylor College of Medicine, Houston, Texas 77030, USA;; 6 Human Genome Sequencing Center, Baylor College of Medicine, Houston, Texas 77030, USA;; 7 Center for Precision Health, McWilliams School of Biomedical Informatics, University of Texas Health Science Center at Houston, Houston, Texas 77030, USA;; 8 Texas Children's Hospital, Houston, Texas 77030, USA;; 9 Cain Pediatric Neurology Research Laboratories, Jan and Dan Duncan Neurological Research Institute, Houston, Texas 77030, USA;; 10 Division of Neurology and Developmental Neuroscience, Department of Pediatrics, Baylor College of Medicine, Houston, Texas 77030, USA;; 11 Department of Neuroscience, Baylor College of Medicine, Houston, Texas 77030, USA;; 12 McNair Medical Institute, The Robert and Janice McNair Foundation, Houston, Texas 77024, USA;; 13 Baylor Genetics Laboratory, Baylor College of Medicine, Houston, Texas 77021, USA;; 14 Science for Life Laboratory, Department of Immunology, Genetics and Pathology, Uppsala University, 751 85 Uppsala, Sweden;; 15 Department of Computer Science, Rice University, Houston, Texas 77251, USA;; 16 Department of Laboratory Medicine, University of Gothenburg, 413 45 Gothenburg, Sweden;; 17 Department of Clinical Genetics and Genomics, Sahlgrenska University Hospital, 413 45 Gothenburg, Sweden

## Abstract

Chromosomal inversions (INVs) are particularly challenging to detect due to their copy-number neutral state and association with repetitive regions. Inversions represent about 1/20 of all balanced structural chromosome aberrations and can lead to disease by gene disruption or altering regulatory regions of dosage-sensitive genes in *cis*. Short-read genome sequencing (srGS) can only resolve ∼70% of cytogenetically visible inversions referred to clinical diagnostic laboratories, likely due to breakpoints in repetitive regions. Here, we study 12 inversions by long-read genome sequencing (lrGS) (*n* = 9) or srGS (*n* = 3) and resolve nine of them. In four cases, the inversion breakpoint region was missing from at least one of the human reference genomes (GRCh37, GRCh38, T2T-CHM13) and a reference agnostic analysis was needed. One of these cases, an INV9 mappable only in de novo assembled lrGS data using T2T-CHM13 disrupts *EHMT1* consistent with a Mendelian diagnosis (Kleefstra syndrome 1; MIM#610253). Next, by pairwise comparison between T2T-CHM13, GRCh37, and GRCh38, as well as the chimpanzee and bonobo, we show that hundreds of megabases of sequence are missing from at least one human reference, highlighting that primate genomes contribute to genomic diversity. Aligning population genomic data to these regions indicated that these regions are variable between individuals. Our analysis emphasizes that T2T-CHM13 is necessary to maximize the value of lrGS for optimal inversion detection in clinical diagnostics. These results highlight the importance of leveraging diverse and comprehensive reference genomes to resolve unsolved molecular cases in rare diseases.

Inversions are intrachromosomal segments rotated 180° and inserted back into the genome. These copy-number neutral structural variants (SVs) are characterized by two breakpoint junctions in *cis*, each mapping to the same (paracentric inversion) or distinct chromosomal arms (pericentric inversion). Inversions larger than the resolution limitation of the screening methodology may not be detected due to the need of phasing both junctions in *cis*. This is bioinformatically challenging, particularly in short-read genome sequencing (srGS) analysis, and a feature which makes inversions prone to high false-negative and false-positive rates (Chaisson et al. 2019). Moreover, recurrent inversions formed by nonallelic homologous recombination (NAHR) use segmental duplications (SDs) or other types of highly similar repeats as recombinant substrates (Stankiewicz and Lupski 2002; Dittwald et al. 2013; Carvalho and Lupski 2016) which adds to the challenge of detecting junctions mapping to poor quality regions of the genome (Kidd et al. 2010; Chaisson et al. 2019; Porubsky et al. 2023a).

The clinical impact of inversions ranges from reproductive problems, due to the production of unbalanced gametes during meiosis, to severe monogenic disease. Such events may be underdiagnosed in rare diseases and targeted analysis approaches are often needed (Grochowski et al. 2021; Pagnamenta et al. 2024). We previously demonstrated that 28% of cytogenetically visible inversions are undetected with srGS (Pettersson et al. 2020), suggesting that the breakpoint junctions are located within large stretches of repetitive sequences, which are challenging to resolve using short-read lengths.

Long-read genome sequencing (lrGS) has been shown to improve alignment, enable phasing, and provide better resolution across repetitive regions (Sudmant et al. 2015; Kronenberg et al. 2018; Logsdon et al. 2020). However, inversions with breakpoints mapping to large repeats remain challenging to resolve even with lrGS, underlying the importance of a complete reference genome (Porubsky et al. 2023a,b). The Telomere-to-Telomere assembly of the CHM13 cell line (T2T-CHM13) may provide increased sensitivity in inversion detection due to its increased resolution across repetitive sequences (Nurk et al. 2022; Porubsky et al. 2023a). This reference genome is the first complete reference, adding over 200 megabase pairs (Mbp) of sequence compared to GRCh38 (Nurk et al. 2022). In fact, both GRCh37 and GRCh38 lack information across hundreds of Mbp of regions such as telomeres, centromeres, and other repetitive regions (Church et al. 2011; Schneider et al. 2017; Ameur et al. 2018; Pan et al. 2019; Eisfeldt et al. 2020; Nurk et al. 2022). Often forgotten resources in human genetic analysis are the closely related nonhuman primate genomes chimpanzee (The Chimpanzee Sequencing and Analysis Consortium 2005; Gordon et al. 2016) and bonobo (Mao et al. 2021) that have been fully sequenced, with up to 99% of gaps closed (Mao et al. 2021). Studies show that sequences unmappable after srGS analysis may be present in nonhuman primates (Sherman et al. 2019; Eisfeldt et al. 2020).

The aim of this study is to evaluate lrGS and complete reference genomes in an effort to better resolve chromosomal inversions, in particular those with breakpoints located in repetitive regions. This is crucial for improving the accuracy of clinical diagnostics and understanding the molecular basis of rare diseases.

## Results

Twelve rare inversions affecting Chromosomes 5, 6, 9, 10, 11, 12, 14, 18, and 19 were all initially detected by karyotyping and genome sequenced using various approaches (Table 1).

**Table 1.** Overview of investigated inversions

Case ID	Ascertainment	Karyotype	GRCh37	GRCh38	T2T	Chimpanzee	Bonobo	Sequencing data
RD_P525	Repeated IVF without pregnancy	46,XY,inv(5)(p13q23)	✓	✓	✓	✓	✓	lrGS
P4855_501	Neurodevelopmental disorder, hearing loss, visual impairment, anosmia, hypogonadism	46,XY,inv(6)(p12q16.3)	×	✓	✓	✓	✓	srGS^a^, lirGS^a^, lrGS
P5371_208	Recurrent miscarriages	46,XY,inv(9)(p13q22)	×	×	×	×	×	srGS^a^, lirGS^a^
BH16643-1	Hypotonia, global developmental delay	46,XX,inv(9)(q12q34.3)dn	×	×	✓	×	×	srGS, lrGS, OGM
P4855_106	Family investigation	46,XY,inv(10)(q11q23)pat	×	×	✓	×	×	srGS^a^, lirGS^a^
P4855_208	Neurodevelopmental disorder	46,XY,inv(11)(p11.1q12)mat	×	×	×	×	×	srGS^a^, lirGS^a^, lrGS
RD_P541	Recurrent miscarriages	46,XX,inv(12)(p11.23q13.3)	✓	✓	✓	✓	✓	lrGS, OGM
RD_P549	Family investigation	46,XY,inv(14)(q24q32)	✓	✓	✓	✓	✓	lrGS
P5370_201	Diabetes type II, Hodgkins lymphoma, hearing loss, hypogonadism, retinitis pigmentosa, acanthosis nigricans, beta thalassemia	46,XY,inv(18)(p11.3q11.2)	×	×	×	×	×	srGS^a^, lirGS^a^
RD_P526	Family investigation	46,XX,inv(18)(p11.23q21.1)	✓	✓	✓	✓	✓	lrGS
RD_P542	Repeated IVF without pregnancy	46,XX,inv(19)(p13.2q13.4)	✓	✓	✓	✓	✓	lrGS
RD_P546	Family investigation	46,XY,inv(19)(p13.2?q13.4)	✓	✓	✓	✓	✓	lrGS

The table displays the reference genome in which the inversion breakpoint junctions were mapped (✓) or absent (×), and the data available (short-read [sr], linked-read [lir], long-read [lr] genome sequencing [GS], and optical genome mapping [OGM]).

^a^Previously reported in Pettersson et al. (2020).

### Structural variant calling and filtering

Comparing the amount of SV calls between the srGS and the lrGS, the number of calls is higher in lrGS (average of 14,859 and 7814, respectively, for lrGS and srGS). Overall, SV calling on the lrGS from the inversion carriers aligned to GRCh38 generates more calls in 8/9 cases, compared to alignment to T2T-CHM13 (Supplemental Fig. S1). The number of filtered SV calls rapidly decreases when applying allele frequency filters (Supplemental Fig. S1). The number of SV calls supporting an inversion between the regions known from the cytogenetic analysis ranged from 0 to 2 (Supplemental Table S1).

### Resolving inversions

**Figure 1.**
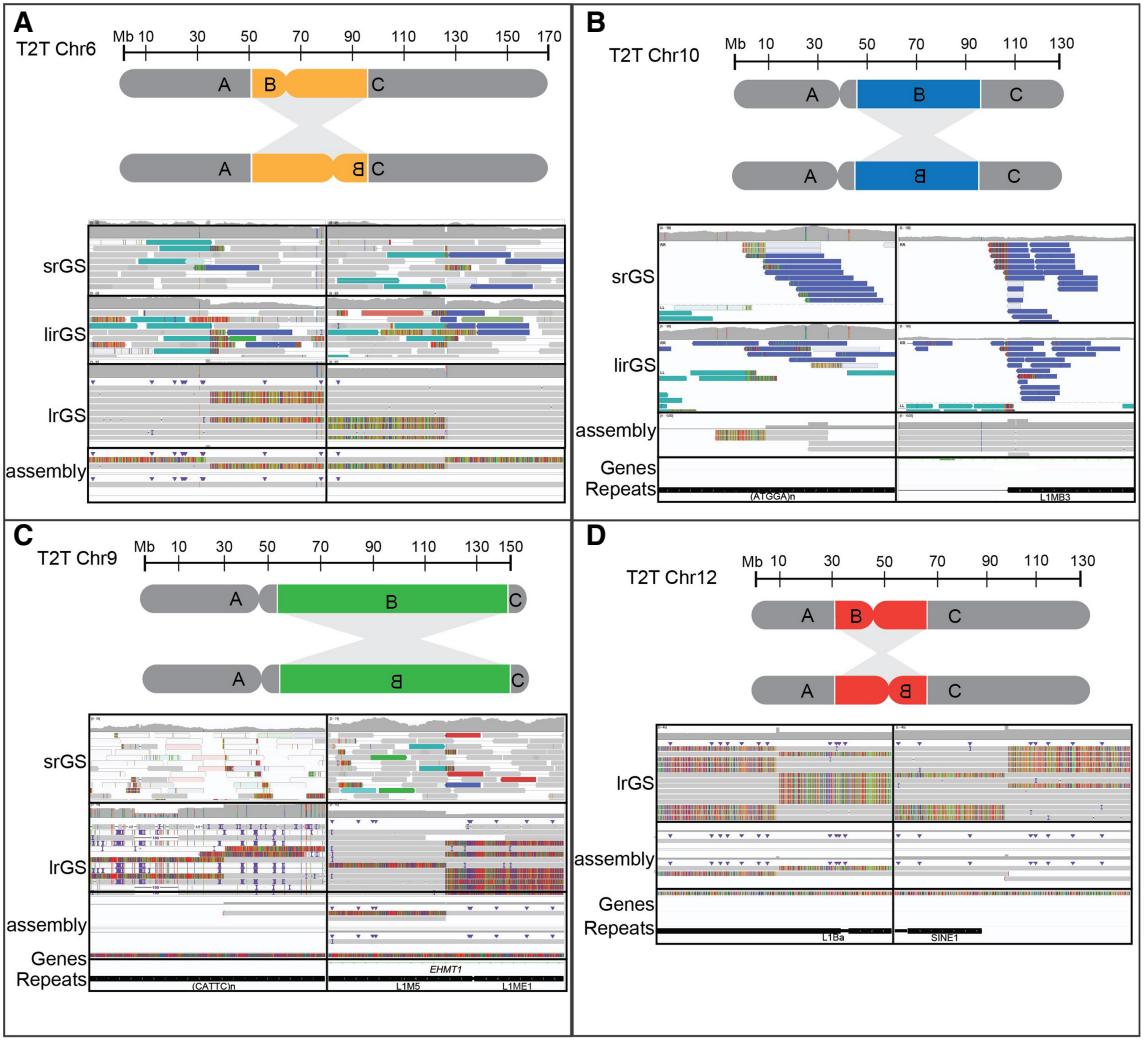
Reference genome-dependent detection of inversions analyzed by srGS and lrGS. (*A*) An inversion 6 (P4855_501) visible in srGS, linked read genome sequencing (lirGS), and lrGS using GRCh38. (*B*) An inversion 10 (P4855_106) visible in srGS and lirGS data using T2T-CHM13. (*C*) An inversion 9 (BH16643-1) only visible by lrGS de novo assembly using T2T-CHM13. (*D*) An inversion 12 (RD_P541) within a 8 kbp DRR.

**Figure 2.**
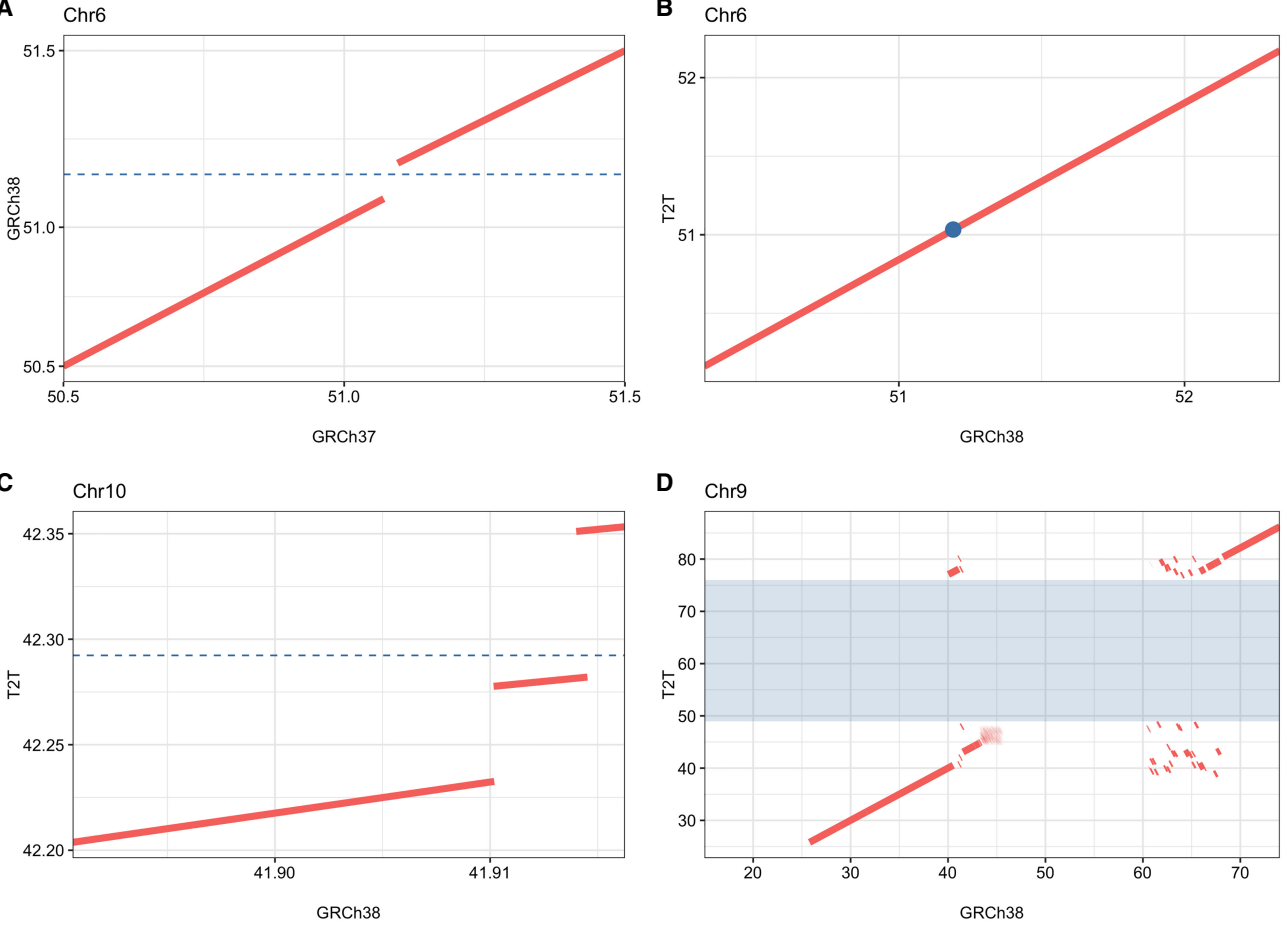
Comparison of the inversion breakpoint region on Chromosome 6p12.3, Chromosome 10q11, and Chromosome 9q12. Reference sequences were aligned with each other and shown as dot plots. The dashed line or dot represents the position of the breakpoint of the inversions. (*A*) The Chromosome 6p inversion breakpoint is located in a 127 kbp region in GRCh38 missing from GRCh37. (*B*) The Chromosome 6p inversion breakpoint in GRCh38 and T2T. (*C*) The Chromosome 10q breakpoint is located in a 69 kbp region missing in GRCh38, with a surrounding 4 kbp duplication which occurs only once in T2T. (*D*) The Chromosome 9q12 breakpoint is located in a 28 Mbp region missing in GRCh38 shaded in blue.

Inversion breakpoints were resolved in 9/12 inversions using both srGS, lrGS, and a reference agnostic approach (Tables 1, 2). The breakpoint junction information along with proposed formation mechanisms for all the nine resolved inversions is provided in Table 2 and Supplemental Figures S2 and S3. Of the nine resolved cases four inversions (P4855_501, P4855_106, BH16643-1, and RD_P541) had one breakpoint junction that was located in regions of the reference which were missing in some genome builds (Figs. 1, 2). One inversion was detected in both GRCh38 and T2T-CHM13 (INV6, P4855_501) and two only in T2T-CHM13 (INV10; P4855_106 and INV9; BH16643-1). The remaining five inversions, all present in individuals referred due to fertility problems, had breakpoints located in easily mappable genomic regions represented in all reference genomes (INV5; RD_P525, INV14; RD_P549, INV18; RD_P526, INV19; RD_P542; and INV19; RD_P546) (Table 1; Supplemental Fig. S3). Three of them had breakpoint junction features consistent with nonhomologous end joining (NHEJ) (INV5; RD_P525) or microhomology-mediated end joining (MMEJ) (INV14; RD_P549, INV18; RD_P526). One case, the INV19 in RD_P542, harbored a 335 bp deletion in one junction indicative of a replicative mechanisms such as microhomology-mediated break-induced replication (MMBIR) and the final case was complex (INV19; RD_P546) (Table 2). The inversions with breakpoint junctions mappable only in specific references as well as the complex rearrangement are detailed below.

**Table 2.** Junction characteristics of identified inversions (T2T-CHM13)

Sample ID	Size (Mbp); % Chr	Chr	PosA	PosB	Microhomology	Insertion	Junction indels pos A	Junction indels pos B	Gene posA	Gene posB	Repeat pos A	Repeat pos B	Likely mechanism jntc1/jnct2
RD_P525	85; 47%	5	42125501	127429118	1	–	5 bp del	8 bp del	*OXCT1* (NM_000436.4), intron 1	*MARCHF3* (NM_178450.5), intron 3	–	L1PA3	NHEJ
			42125495	127429109	–	RIns 32nt Tins 17nt							
P4855_501	43; 25%	6	51032755	94376921	2	–	8 bp dup	2 bp del	–	–	*AluJb*, L1MA9, L1PA3	L1PA4, LTR:MLT1J2	MMEJ
			51032765	94376918	3	–							
BH16643-1	73–101; 53%–73%	9	48424795–77056693	150079672	2	–	–	4 bp del	–	*EHMT1* (NM_001354263.2), intron 25	(ATTC)n	L1M5	MMEJ
			48424795–77056693	150079676	3	–							
P4855_106	53; 40%	10	42197576–42315905	96022615	–	–	–	14 bp del	–	–	(ATGG)n, (ATGGA)n, (TGGAA)n	L1MB3	NHEJ
			42197576–42315905	96022600	–	–							
RD_P541	25; 19%	12	32945545	58051150	2	–	6 bp dup	6 bp dup	–	–	Tigger1, L1Ba	–	MMEJ
			32945540	58051144	1	–							
RD_P549	34; 32%	14	63951601	97962156	3	–	8 bp del	5 bp dup	*SRSF5*, intron 1	*ZFYVE21* (NM_001198953.2), intron 1	L1MC5a, *AluY*, MER4A1	–	MMEJ
			63951610	97962152	1	–							
RD_P526	41; 50%	18	7340601	47883888	2	–	2 bp dup	2 bp dup	–	*LOC*105372100	–	MIR/L2	MMEJ
			7340602	47883889	2	–							
RD_P542	48; 82%	19	9982025	58312124	–	TIns 23nt	1 bp dup	335 bp del	*OLFM2* (NM_001304347.2), intron 5	*AC010327.5*, intron 1	L2b	(AT)n, *AluJb*, LT1B	MMBIR
			9982025	58312461	2	–							
RD_P546	49; 84%	19	3256970	56644686	–	RIns 90nt	–	–	*CELF5* (NM_001172673.2), intron 6	*ZNF331* (NM_018555.6), intron 2	L1ME3	L2b	Complex MMBIR
			56603650	61454533	2	–	–	–	*ZNF331* (NM_001317120.2), intron 1	*ZNF497* (NM_001207009.2), intron 1	L1PA6	–	
			11999009	61440737	–	RIns 61nt	–	–	*ZNF439*, intron 1	–	–	*AluY*, L1MEf	
			3281277	6700567	–	RIns 18nt	–	–	*AC010649.1,* intron 1	*C3,* (NM_000064.4), exon 12	SINE/MIRB	–	
			6755880	12044892	–	RIns 16nt	–	–	*SH2D3A*, (NM_001386583.1), intron 1	*ZNF69*	L2a	MER92b;LTR	

Each row represents a junction, and characteristics such as size, junction dup/dels, genes, repeats, insertions, and microhomology are given.

(FoSTeS/MMBIR) fork stalling and template switching/microhomology-mediated break-induced replication, (NHEJ) nonhomologous end joining, (MMEJ) microhomology-mediated end joining, (NAHR) nonallelic homologous recombination, (RIns) random insertion, (Tins) templated insertion.

Case P4855_501 suffered hearing impairment, intellectual disability, autistic features, diplopia, anosmia as well as hypogonadism and had a 43 Mbp pericentric inversion on Chromosome 6 that was not detected using srGS (Pettersson et al. 2020), lrGS and de novo assembly in GRCh37. Inversion breakpoint junctions were located upon alignment to GRCh38 and T2T-CHM13, where it could be detected in srGS, lrGS, and de novo assembly (Fig. 1A). In fact, the inversion breakpoints were detectable also by alignment to the chimpanzee and bonobo references (Supplemental Figs. S4, S5). This was due to a 127 kbp gap in GRCh37 at 6p12.3 which was present in all the other human and primate reference genomes (Fig. 2A,B). The region, located at Chr 6: 51,102,785–51,230,413 (GRCh38) did not contain any known genes and consists of 51% repeat sequence; 49% interspersed repeats, and 2% simple repeats (Fig. 6C). Discordant reads pairs in srGS and split reads in lrGS were present in the GRCh37 alignment at the 6q16.1 breakpoint, partnering with multiple genomic locations (Supplemental Fig. S4). Both breakpoint junctions contained microhomology (2–3 bp) indicating MMEJ (Table 2). No genes were interrupted by the inversion breakpoints and 324 genes were located within the inverted segment. By analyzing topological associated domains (TADs) in public data sets from 3D genome browser (Dixon et al. 2012), we find multiple TADs that merge or fall closer to each other due to the inversion, but none of these clearly explain the clinical symptoms (Supplemental Fig. S6A).

Case P4855_106, a healthy male whose partner had repeated miscarriages had a ∼53 Mbp inversion on Chromosome 10, undetected by srGS and linked read genome sequencing (lirGS) in GRCh37 (Pettersson et al. 2020). No DNA was available for lrGS, but the inversion was identified in T2T-CHM13 (Fig. 1B; Supplemental Fig. S2), where it was visible by both srGS and de novo lirGS assembly. The 10q11 breakpoint, located in a region containing simple repeats, could only be pinpointed to a span at Chr 10: 42,197,576–42,315,905 (T2T-CHM13). Overlapping this was a 69 kbp region of simple repeats only present in T2T-CHM13 (Fig. 2C; Supplemental Fig. S5). The region, spanning from Chr 10: 42,282,056–42,351,085 (T2T-CHM13) does not contain any known genes and consists of 99% simple repeats and is surrounded by other regions of simple repeats. The breakpoint junctions did not show any microhomology but a 14 bp deletion indicating NHEJ (Table 2). The inversion does not interrupt any known genes, and 2879 genes are located within the inverted segment. TAD analysis shows that TADs identified in multiple tissues are broken, merge, or fall closer to each other due to the inversion (Supplemental Fig. S6B).

Case RD_P541 with repeated miscarriages had a 25 Mbp inversion on Chromosome 12 (Table 1; Fig. 1D). The initial lrGS analysis included both reference and de novo assembly using GRCh37, GRCh38, and T2T-CHM13. The detailed analysis revealed the 12q14.1 breakpoint was located in an 8844 bp region only present in T2T-CHM13, chimpanzee, and bonobo (Supplemental Fig. S7), which could not have been bridged by srGS. The region consists of 66% repeats, determined to be an L1 element. The gap was spanned by both lrGS as well as optical genome mapping (OGM) (Supplemental Fig. S8). The breakpoint junctions did not show any microhomology, but short (6 bp) duplications were present on both sides indicating MMEJ (Table 2). The inversion does not break any known genes, but TADs identified in multiple tissues were broken and merged due to the inversion (Supplemental Fig. S6C).

**Figure 3.**
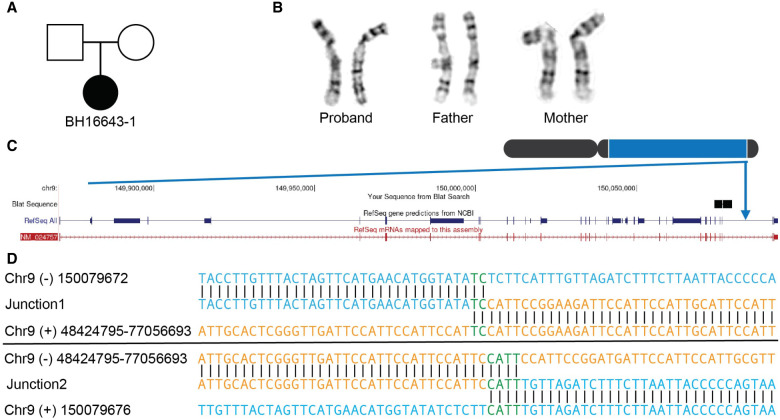
Inversion affecting Chromosome 9 (BH16643-1). (*A*) Pedigree displaying inheritance pattern for inversion 9. (*B*) G-banded chromosome analysis showed a paracentric inversion in the long arm of one Chromosome 9 between bands 9q12 and 9q34.3 in the proband. The abnormal Chromosome 9 is to the *right*. Parental chromosome analysis revealed no evidence of this inversion in either parent, suggesting that this is a de novo event. (*C*) Chromosome 9 inversion disrupted intron 25 out of 26 of *EHMT1* at 9q34.3. (*D*) Nucleotide sequence alignment of inversion breakpoint junctions 1 (*top*) and 2 (*bottom*).

Case BH16643-1 with global developmental delay, hypotonia, feeding difficulties, congenital heart defect, and dysmorphic craniofacial features had a ∼95 Mbp inversion on Chromosome 9 (Figs. 1C, 3A,B; Supplemental Fig. S9; Supplemental Document 1). The inversion was undetected in srGS, lrGS, and OGM using GRCh37. Manual inspection of the OGM data indicated an SV breakpoint junction at 9q34.3 that was narrowed down to 150.05–150.1 Mbp using T2T-CHM13 OGM de novo assembly (Supplemental Fig. S9). Using T2T-CHM13, OGM, lrGS, and de novo assembly, we were able to pinpoint the 9q34.3 breakpoint. The 9q12 breakpoint was located in a 28 Mbp region (Chr 9: 48,424,795–77,056,693, T2T-CHM13) consisting of 19% satellite and 79% simple repeats not represented in reference genomes GRCh37 and GRCh38 nor in bonobo or chimpanzee (Figs. 2D, 5A). Due to the high repeat level, the 9q12 breakpoint is ambiguously aligned in both OGM, lrGS, and de novo assembly contigs (Fig. 1C). Regardless, breakpoint sequence analysis was possible, revealing presence of 2–3 bp microhomology (Fig. 3D; Table 2) suggesting an MMEJ mechanism. The 9q34 breakpoint interrupts intron 25 of the gene *EHMT1* (Fig. 3C), haploinsufficiency of which causes Kleefstra syndrome 1 (MIM#610253), a diagnosis fitting the clinical phenotype (Supplemental Document 1). RNA sequencing revealed a lower expression of *EHMT1* (fold change = 0.56, *Z*-score = −5.54, *P*-adjusted = 0.06) (Supplemental Fig. S10).

**Figure 4.**
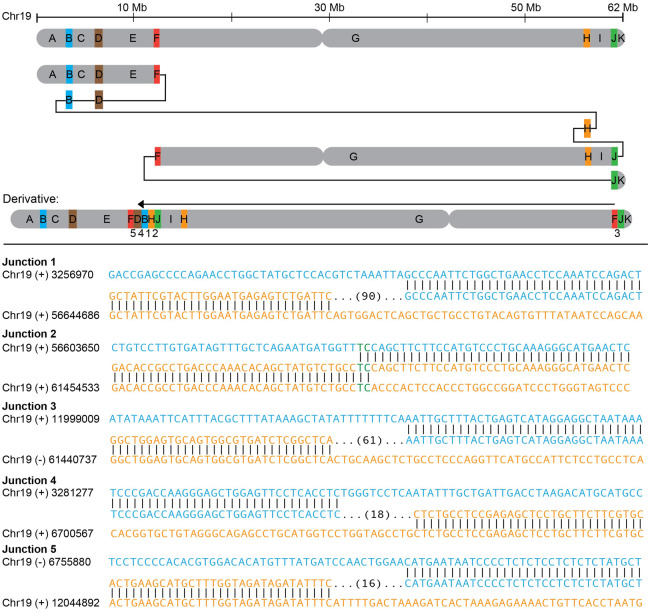
Complex inversion on Chromosome 19 (RD_P546). (*Upper* panel) Inversion structure with duplicated segments in color and nonduplicated segments in gray. Junction numbers are given below the resulting derivative. (*Lower* panel) Breakpoint junction sequences with number of base pairs inserted in parentheses.

An INV19 (RD_P546) was revealed to be a part of a complex rearrangement involving five duplications as well as an inversion (Fig. 4). The small duplications (median size of ∼40 kbp) were spread across 49 Mbp on Chromosome 19 and show patterns constant with MMBIR (Table 2). The specific individual was investigated due to fertility problems and was otherwise healthy.

### Comparing variable sequences in human and primate reference genomes

Our analysis revealed that inversion breakpoints seem to cluster in regions of the genome that are poorly characterized in some human reference genomes. This impact of reference genomes on clinical SV calling prompted us to investigate the prevalence and population frequencies of such differential reference regions (DRRs), that is, sequences larger than 10 kbp that are present in one reference and missing in another.

DRRs were identified by pairwise comparison of three human reference genomes (GRCh37, GRCh38, and T2T-CHM13) and two primate reference genomes (chimpanzee and bonobo) reference genomes. In these comparisons, the longest uninterrupted DRR was found between GRCh38 and GRCh37 (10 kbp–47 Mbp, median 50 kbp), while the most fragmented DRRs were observed between T2T-CHM13 and GRCh38 (10 kbp–34 Mbp, median 30 kbp). The chimpanzee-T2T-CHM13 comparison showed DRRs ranging from 10 kbp to 14 Mbp (median 40 kbp), and the bonobo-T2T-CHM13 comparison had DRRs ranging from 10 kbp to 19 Mbp (median 35 kbp). In total, we identified 203 regions spanning 260.6 Mbp that are present in T2T-CHM13 and missing from GRCh37 (T2T-GRCh37). Notably, T2T-GRCh37 contains the highest total Mbp of DRRs (Table 3; Supplemental Table S2).

**Table 3.** Differential reference regions between reference genomes

	Query					
	DRR (Mbp)	GRCh37	GRCh38	T2T	Chimpanzee	Bonobo
Template	GRCh37	0	8.5	12.6	52.5	59.7
		0	84	130	686	717
	GRCh38	81.03	0	39.44	117.8	125.5
		340	0	814	870	885
	T2T	260.6	216.9	0	289.36	295.3
		203	687	0	878	922
	Chimpanzee	333.2	325.1	315.1	0	263.9
		743	827	845	0	849
	Bonobo	408.9	400.7	392.48	336.1	0
		829	877	855	992	0

For each template on the *top* row, the total amount of sequence in megabase pairs (Mbp) and on the *second* row, the total number of DRRs is given in comparison with the query reference.

**Figure 5.**
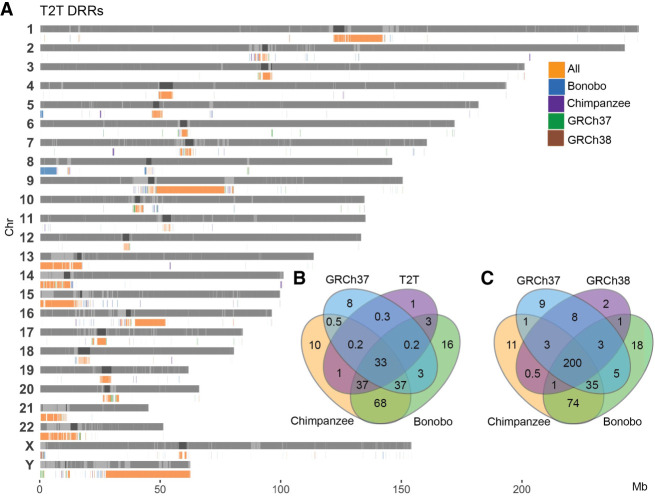
Shared DRR in T2T-CHM13 and GRCh38. (*A*) Bar plot of all T2T DRRs, (*B*) Venn diagram of Mbp overlap between all GRCh38 DRRs, and (*C*) Venn diagram of Mbp overlap between all T2T DRRs.

When comparing all DRRs where a sequence was present in T2T-CHM13 and missing from the query genome (T2T DRRs), we observe clustering of DRRs located in centromeric and telomeric regions as well as SDs, the acrocentric p-arms, and Chr Y (Fig. 5A). Of all T2T DRRs, 200 Mbp of the sequence was missing from all query reference genomes (Fig. 5C). For all GRCh38 DRRs, only 33 Mbp of the sequence was missing in all query reference genomes including T2T-CHM13 (Fig. 5B; Supplemental Fig. S11).

### DRRs introduce repetitive sequences

**Figure 6.**
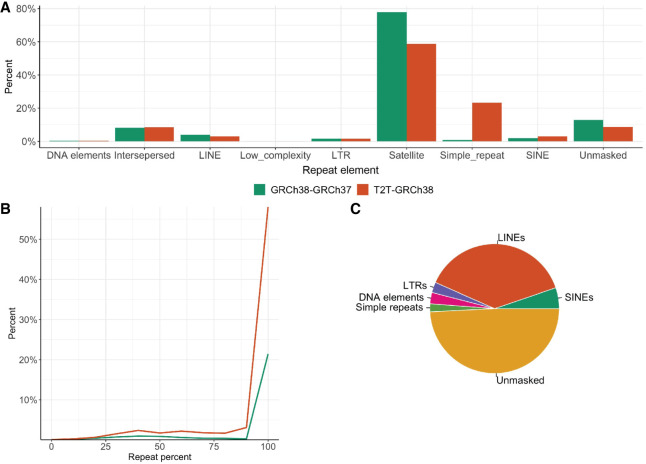
Repeat characterization across DRRs. (*A*) Percentage of repeat elements (masked by RepeatMasker) in the DRR sequences from GRCh38–GRCh37 and T2T-GRCh38. (*B*) Distribution of DRR sequences and their repeat percentage in GRCh38–GRCh37 and T2T-GRCh38. (*C*) Pie chart displaying repeat content in the GRCh38–GRCh37 DRR sequences affected by the inversion 6 at the 6p12 junction in GRCh38.

Inversions are known to be associated with repeat sequences (Stankiewicz and Lupski 2002; Dittwald et al. 2013; Carvalho and Lupski 2016). Of the four DRRs involved in inversions reported in this article, 2/4 had a repeat level >80%. Only the 127 kbp DRR affected by the inversion on Chromosome 6 consisted of 49% unmasked sequence, and 51% repeats; 38% long interspersed nuclear elements (LINEs), 5.2% short interspersed nuclear elements (SINEs), 2% simple repeats, 2.6% long terminal repeats (LTRs), and 2.8% DNA elements (Fig. 6C). Repeat analysis of all DRRs in GRCh38–GRCh37 and T2T-GRCh38 reveal most to be repeat regions, and ∼10% to be unique sequence (Fig. 6A). Of all T2T-GRCh38 DRR sequences, 55% consisted of 100% repetitive DNA, 20% were located inside or within 10 kbp of centromeric regions, and 30% within SDs (Fig. 6B). Of GRCh38–GRCh37 DRR sequences, 20% consisted of 100% repetitive DNA, 76% were located within 10 kbp of centromeric regions, and 20% within SDs (Fig. 6B).

### DRR sequences in the general population

Next, we wanted to look further into the DRRs and their distribution in a population data set. We aligned srGS data from 100 Swedish individuals (Ameur et al. 2017) to the five references and assessed the coverage across the DRR for the population (Supplemental Figs. S12–S14).

Of the DRRs in T2T-GRCh38, 68% were classified as not detected, meaning that the average coverage per individual was below the cutoff of 8× (Supplemental Fig. S12; Supplemental Table S3). Of the DRRs detected (32%), 42% were observed in <5% (rare), 58% were found in >5% (common), and 30% in over 90% (Supplemental Fig. S12C). Across the 100 individuals, an average of 1.3% of reads spanning DRRs were multimapping reads, meaning they map to several locations in the genome. We also assessed the mapping quality of reads from five individuals across DRRs (Supplemental Fig. S15) where 20% of reads had a mapping quality above 20.

In comparison, for the GRCh38–GRCh37 DRRs, 60% were not detected (Supplemental Fig. S14). Of the detected DRRs (40%), 13% were rare, and 86% were common. Furthermore, 53% were found in over 90% of the queried individuals. The mapping quality of reads from five individuals across DRRs was assessed, where 25% of reads had a mapping quality above 20 (Supplemental Fig. S15). The violin plot confirms that most DRRs lack aligned reads (Supplemental Fig. S12).

## Discussion

The availability of lrGS and T2T-CHM13 prompted us to revisit five previously unsolved and seven novel cytogenetically visible inversions, successfully resolving nine of them (75%). Notably, in four cases, one inversion breakpoint region was missing from one of the human reference genomes GRCh37 or GRCh38, necessitating an analysis with T2T-CHM13. One case required lrGS and de novo assembly for resolution. This illustrates how reference genome variability may influence the accuracy of clinical diagnostic SV calling and that lrGS alone cannot overcome reference genome flaws.

Combining our data with previous work (Pettersson et al. 2020; Grochowski et al. 2021), out of a total of 26 cytogenetically visible inversions, we were able to molecularly resolve 23/26 (88%) and are still missing 12%. We have suggested that those missing cytogenetically visible inversions form through NAHR explaining why some remain undetected even after srGS analysis (Stankiewicz and Lupski 2002; Kidd et al. 2008; Carvalho and Lupski 2016; Pettersson et al. 2020). However, breakpoint junction analysis of the nine inversions resolved here shows that they are not mediated by ectopic recombination between paralogous sequences but formed through NHEJ, MMEJ, and replicative mechanisms (Table 2). Hence, the contribution of NHEJ and MMEJ to the formation of inversions is ∼65% (15/23). Furthermore, complexities and unbalances >100 bp at the breakpoint junctions were observed in two of the nine inversions, and combined with the previous data seem to be a common phenomenon in cytogenetically detected inversions (26%, 6/23). This result supports that cytogenetically balanced inversions, like translocations, may result from an error prone repair of processed double-strand breaks (DSBs) (Nilsson et al. 2017). In contrast, common polymorphic inversions are typically formed through NAHR (Stankiewicz and Lupski 2002; Abergel et al. 2007; Porubsky et al. 2023a), indicating a correlation between population frequencies and formation mechanisms.

In BH16643-1 (INV9), the lrGS and T2T-CHM13 analysis resulted in a molecular diagnosis. One breakpoint disrupted *EHMT1* likely leading to loss of function of the gene, consistent with the expected underlying biological mechanism for Kleefstra syndrome 1 (MIM#610253), as supported by the RNA results. We previously reported a patient with multiple paracentric and pericentric copy-neutral inversions affecting Chromosome 6, disrupting *ARID1B* in an individual with a neurodevelopmental phenotype (Grochowski et al. 2021). These results underscore the relevance of inversions to unsolved rare disease, which are often undetected by current clinical genome sequencing.

Our studies of T2T-CHM13 DRRs show that most of the added sequence compared to GRCh38 consist of repetitive DNA (Fig. 6). Resolving variants in repeat regions is challenging, especially using srGS, as exemplified by the INV9 (BH16643-1) where lrGS with de novo assembly was necessary to identify a breakpoint located in the highly repetitive 9q12 region. Still, the 9q12 breakpoint could not be fully pinpointed. Additionally, OGM was an asset in this case, as well as for INV12, with the abilities to identify large genomic rearrangements. However, even here, the lack of labels in challenging regions in the genome limits our findings (Supplemental Fig. S9). The repetitiveness of the DRRs can also explain the SweGen srGS results, where reads aligned to T2T-CHM13 DRRs had a low mapping quality (20% with a mapping quality >20) (Supplemental Fig. S15).

Regardless, INV10 (P4855_106) residing in a region consisting of 99% simple repeats could be detected using srGS; however, the details of the 10q11 breakpoint would need further refinement using lrGS. These results indicate that the detection of a true positive SV call is highly dependent on the completeness of the reference as well as the representation of normal variation, especially using srGS, but even when applying lrGS. This is important from a clinical perspective, where lrGS, which improves the resolution of repeats, is not yet broadly available. As an exception, the INV6 127 kbp (P4855_501) and INV12 8 kbp (RD_P541) DRRs only contained 51% and 66% repeat sequence, like other regions of the genome (Nurk et al. 2022). Notably, both these regions were present in the chimpanzee (The Chimpanzee Sequencing and Analysis Consortium 2005) and bonobo (Mao et al. 2021), highlighting that these reference genomes can add genomic diversity to our present references.

Our results (216 Mbp and 260 Mbp DRR in T2T-CHM13 compared to GRCh38 and GRCh37, respectively) are comparable to previous work showing that T2T-CHM13 introduce >200 Mbp compared to GRCh38 (Schneider et al. 2017; Nurk et al. 2022) indicating that the T2T reference is more complete. Although we now have an almost 100% fully resolved human reference genome, no single genome can represent the full genetic diversity in humans. To address these shortcomings, the pangenome consortium made a reference genome representing 47 diploid assemblies represented as a graph (Liao et al. 2023). This assembly can represent large genomic variation, complex loci, and increased number of SVs per haplotype. With future refinement and aspects of including >700 haplotypes, this will provide a better representation of the human genome. Along with samples representing the local population, this approach could provide better alignment and variant calling (Ten Berk de Boer et al. 2024).

In conclusion, lrGS shows great promise in advancing clinical SV analysis. However, solving of rearrangements in variable genomic regions depends heavily on the reference genome and its completeness. Therefore, novel lrGS databases and verification methods are needed. To fully understand lrGS findings and offer digital karyotyping as a first-line test, we must understand the limits of the analysis. Furthermore, our results highlight that to improve clinical genomic analysis genomic diversity needs to be considered. The available human and primate genomes are a valuable resource to improve our understanding of repetitive and complex regions which have previously been understudied.

## Methods

### Study participants

Eleven inversion carriers were enrolled at Karolinska University Hospital, Stockholm, Sweden. Of these individuals, five (P4855_501, P5371_208, P4855_106, P4855_208, and P5370_201) had previously been analyzed with srGS and lirGS without results (Pettersson et al. 2020) and six individuals (RD_P525, RD_P541, RD_P549, RD_P526, RD_P542, and RD_P546) were newly enrolled. Patient BH16643-1 was enrolled using research protocol H-47281/Pacific Northwest Research Institute WIRB #20202158 and 15HG0130 with the NIH IRB as part of the Undiagnosed Diseases Network (UDN), Baylor College of Medicine. Whole blood samples (3–10 mL) were collected from the patient and parents.

### Ethics approval and consent

For samples P4855_501, P5371_208, P4855_106, P4855_208, and P5370_201, ethical approval was given by the Regional Ethical Review Board in Stockholm, Sweden (ethics permit numbers 2012/222-31/3 and 2019-04746). This ethics permit allows for the use of clinical samples for analysis of scientific importance as part of clinical development. The IRB approval does not require us to get written consent for clinical testing. For samples RD_P525, RD_P541, RD_P549, RD_P526, RD_P542, and RD_P546, ethical approval was given by the Ethical Review Board in Sweden approved the study (ethics permit number 2019-04746). Written consent to participate and publish was provided by the subject or their legal guardians. Patient BH16643-1 was enrolled using research protocol H-47281/Pacific Northwest Research Institute WIRB #20202158 and 15HG0130 with the NIH IRB as part of the Undiagnosed Diseases Network (UDN). Written informed consent to participate and publish was obtained from the legal guardians.

The research conformed to the principles of the Helsinki Declaration.

### Public data sets

The SweGen data set (*n* = 1000) (Ameur et al. 2017), consists of 1000 unrelated Swedish individuals representing the genetic variation in the Swedish population. In brief, the individuals were selected from the Swedish Twin Registry, a nationwide cohort of 10,000 Swedish-born individuals. The samples were sequenced using Illumina short-read sequencing to an average of 30× coverage. From these, we selected 100 unrelated samples for further use in this study.

### Genome sequencing

For samples (P4855_501, P5371_208, P4855_208, P4855_106, and P5370_106) srGS and 10x Genomics lirGS was performed as singletons at the National Genomics Infrastructure (NGI) at Science for Life Laboratory (SciLifeLab) Stockholm as previously mentioned (Pettersson et al. 2020).

lrGS was performed using Pacific Biosciences (PacBio) Sequel II (P4855_501, P4855_208) or Revio (RD_P541, RD_P525, RD_P526, RD_P542, RD_P546, and RD_P549) with one SMRTcell per sample, to an average read length of 16.4 kbp (NGI SciLifeLab).

For the BH16643 family, short-read trio genome sequencing was performed at the Baylor College of Medicine Human Genome Sequencing Center (HGSC) with KAPA Hyper PCR-free reagents on the NovaSeq 6000 to an average of 37× coverage. Postsequencing data analysis was performed using the HGSC HgV analysis pipeline (Regier et al. 2018). lrGS of the trio was done on the PacBio Sequel II instrument using two SMRTcells.

### Genome analysis

The srGS and lirGS data were aligned to reference genomes GRCh37, GRCh38, T2T, chimpanzee, and bonobo using BWA-MEM (Li and Durbin 2009) and Long Ranger (Marks et al. 2019), respectively (Supplemental Code).

Variant calling was performed using FindSV as described previously (Pettersson et al. 2020). FindSV allows for annotation of variant frequency using a database, where an in-house database was used (*n* = 1000 for GRCh38 and *n* = 100 for T2T).

The lrGS data were aligned to GRCh37, GRCh38, and T2T. Analysis was done using an in-house developed pipeline LOMPE (https://github.com/kristinebilgrav/LOMPE). LOMPE uses minimap2 for alignment and combines Sniffles (v1) (Sedlazeck et al. 2018) and CNVpytor (Suvakov et al. 2021) for SV calling, and produces a single VCF file which is annotated using variant effect predictor (VEP) (McLaren et al. 2016). Additional annotation of population frequency is performed with an in-house database (*n* = 10 for GRCh38 and *n* = 5 for T2T) and SVDB (Eisfeldt et al. 2017). The resulting lrGS data had a read depth of 24 (inv5), 13 (inv6), 26 (inv9), 10 (inv11), 18 (inv12), 23 (inv14), 27 (inv18), 25 (inv19), and 27X (inv19) and an average read length of 18 kbp (Hinrichs et al. 2006).

### De novo assembly

De novo assembly using lrGS from samples P4855_501, P4855_208, and RD_P541 was performed using hifiasm (Cheng et al. 2022) (Supplemental Code). For sample BH16643-1, trio-binned assemblies were produced using yak (https://github.com/lh3/yak) and hifiasm (Cheng et al. 2022). Quality control was performed using quast (Mikheenko et al. 2018). Alignment to reference genomes GRCh37, GRCh38, and T2T was performed using minimap2 (Li 2018), and variant calling was performed using SVIM-asm (Heller and Vingron 2021). On lirGS from sample P4855_106 a de novo assembly was performed using 10x Genomics Supernova (Weisenfeld et al. 2017).

### RNA sequencing

Transcriptome analysis was conducted similar as previously described (Murdock et al. 2021). Briefly, RNA from skin fibroblasts was quantified and processed using a stranded, poly(A)-tailed kit (Illumina) before being multiplexed and subjected to 150 bp paired-end sequencing with ∼150 million reads generated per sample. Aberrant expression events were detected by Detection of RNA Outlier Pipeline (DROP) (Yépez et al. 2021) using the default, recommended settings for OUTRIDER (Brechtmann et al. 2018).

### Optical genome mapping

OGM of sample BH16643-1 was performed as described previously (Grochowski et al. 2024). Briefly, ultra-high molecular weight (UHMW) genomic DNA for use in genomic optical mapping was extracted from blood using Bionano Prep Blood and Cell Culture DNA Isolation Kit (Bionano Genomics) with an input of 1.5 million cells. Subsequent DNA quantity and size were confirmed using Qubit dsDNA BR Assay Kit. A total of 0.75 µg of HMW DNA was labeled by DLE-1 using the Bionano Prep direct label and stain (DLS) method (Bionano Genomics) and loaded onto a flow cell to run on the Saphyr System (Bionano Genomics). Raw optical mapping molecules in the form of BNX files were run through a preliminary bioinformatic pipeline that filtered out molecules <150 kbp in size and with <9 motifs per molecule to generate a de novo assembly of the genome maps. The data collected provided 1637 Gbp of data >150 kbp, with at least nine labels per molecule. Data were then aligned to an in silico reference genome (GRCh37, GRCh38, and T2T-CHM13) using the Bionano Solve v3.7 RefAligner module. SV calls were generated through a comparison of the reference genome using a custom Bionano SV caller. Manual inspection of proposed breakpoint junctions was visualized in the Bionano Access software program v1.7.2.

For RD_P541, UHMW DNA was extracted from frozen EDTA-blood using SP Blood and Cell Culture DNA Isolation Kit v2 (Bionano Genomics) and following SP Blood and Cell Culture DNA Isolation Protocol v2 (document no. 30398, revision B). The UHMW DNA was labeled and stained using the Bionano Prep Direct Label and Stain Generation 2 (DLS-G2) kit (Bionano Genomics) with the corresponding protocol (document no. 30553-1, revision D). The labeled HMW DNA was loaded on a chip, and subsequently captured and analyzed in the Saphyr instrument (Bionano Genomics) and Bionano Solve v3.8. The interpretation was performed in Bionano Access software v1.8.

### TAD analysis

BED files containing TADs identified in the hippocampus, cortex, epidermal cells, prostate cells, and aorta were downloaded from http://3dgenome.fsm.northwestern.edu/ (Dixon et al. 2012) and visualized using the Integrative Genomics Viewer (IGV) (Robinson et al. 2011). BED files with TADs in GRCh38 were converted into T2T coordinates using UCSC liftOver (Hinrichs et al. 2006).

### Reference genome analysis

Reference genomes GRCh37 (GCF_000001405.13), GRCh38 (GCF_000001405.26), T2T-CHM13 (v2.0, GCF_009914755.1), bonobo (GCF_029289425.1), and chimpanzee (GCF_028858775.1) were downloaded from National Center for Biotechnology Information (NCBI) (Sayers et al. 2022). Alternative sequences were excluded for all reference genomes. They were aligned to one another using minimap2 using the settings for cross-species full genome alignment and overlap between long reads (2.24-r1122) (Li 2018; Supplemental Code). This enables sequence comparison between the two reference genomes.

Coverage analysis of the resulting pairwise compared reference genomes was performed using TIDDIT v.3.6.0 (Eisfeldt et al. 2017), producing a BED file with gaps between the query and template. Files with known gap regions were downloaded from UCSC Table Browser (Karolchik 2004) and these regions were excluded from the coverage analysis. A DRR was identified as a region of the template genome which was not covered by the query genome.

Repeat content within DRRs was assessed using RepeatMasker (Smit et al. 2013).

### Differential reference regions in SweGen

One hundred SweGen (Ameur et al. 2017) samples were aligned to each of the five reference genomes and coverage analysis across the genome was performed as described above. Coverage across DRRs identified above was extracted. A DRR was considered present in SweGen if the coverage across the DRR >8× and <100×, and absent if the coverage was <8×. Regions with coverage >100× were not considered. The thresholds were set based on coverage experienced to support the presence of one or multiple genomic copies (Supplemental Table S1). On a populational level, a DRR was considered common if it was present in >5% of the population and absent if none had it (Supplemental Code).

For the VENN diagrams, a DRR was considered overlapping if the region was missing in all query genomes, but present in the template genome.

Multimapping reads were identified by extracting the number of times a read was aligned in the BAM file. Mapping quality was assessed by extracting the mapping quality of all reads in the BAM file (Supplemental Code).

## Data access

The raw lrGS data from patient BH16634-1 have been submitted to the NCBI BioProject database (https://www.ncbi.nlm.nih.gov/bioproject/) under accession number PRJNA1092654. The raw lrGS data for patients RD_P525, RD_P541, RD_ P549, RD_P526, RD_P542, RD_P546 have been submitted to the European Genome–phenome Archive (EGA; https://ega-archive.org) under accession number EGAS50000000436. Custom scripts and code used in the analysis can be found in Supplemental Code.

## Supplemental Material

Supplement 1
