# Supplementary material for: Leveraging the T2T assembly to resolve rare and pathogenic inversions in reference genome gaps
Source: Genome Res. 2024 Nov;34(11):1785–97. doi: 10.1101/gr.279346.124 (PMC11610578; doi:10.1101/gr.279346.124)
Supplement: Supplement 1 [file Supplemental_Information.pdf]

Supplemental Information

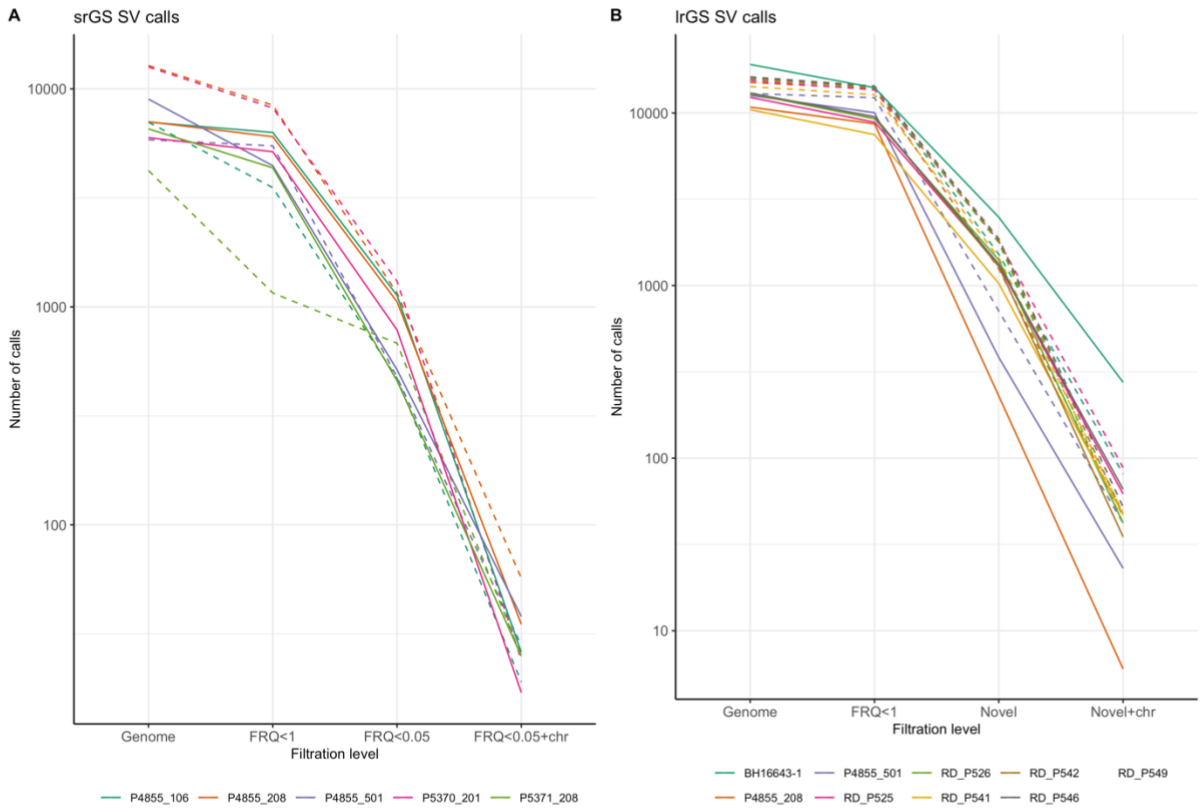

Supplemental Fig. S1: Filtration of SV calls using in-house databases in **A)** srGS and **B)** lrGS. dashed lines GRCh38, full lines T2T. Number of calls in log10 scale.

Supplemental Table S1. Number of SV calls per individual in T2T-CHM13 aligned genomes and *de novo* genomes.

Case ID	Cytogenetic abberation	Genome	Affected chr	Cytoband A	Cytoband B	A-B match
RD_P525	inv(5)(p13q23)	12321	659	128	39	1
P4855_501	inv(6)(p12q16.3)	12636	503	42	32	1
P4855_501 *de novo*	inv(6)(p12q16.3)	37127	1579	189	83	2
BH16643-1	inv(9)(q12q34.3)	19105	1888	1524	86	0
BH16643-1 *de novo*	inv(9)(q12q34.3)	41400	3091	2237	235	1
P4855_106*	inv(10)(q11q23)	20068	515	129	54	2

RD_P541	inv(12)(p11.23q13.3)	10449	482	105	56	1
RD_P541 *de novo*	inv(12)(p11.23q13.3)	37551	1524	488	130	2
RD_P549	inv(14)(q24q32)	12513	466	45	107	1
RD_P526	inv(18)(p11.23q21.1)	12942	451	133	50	1
RD_P542	inv(19)(p13.2q13.4)	13021	574	42	91	1
RD_P546	inv(19)(p13.2?q13.4)	13050	483	44	94	1

*srGS data

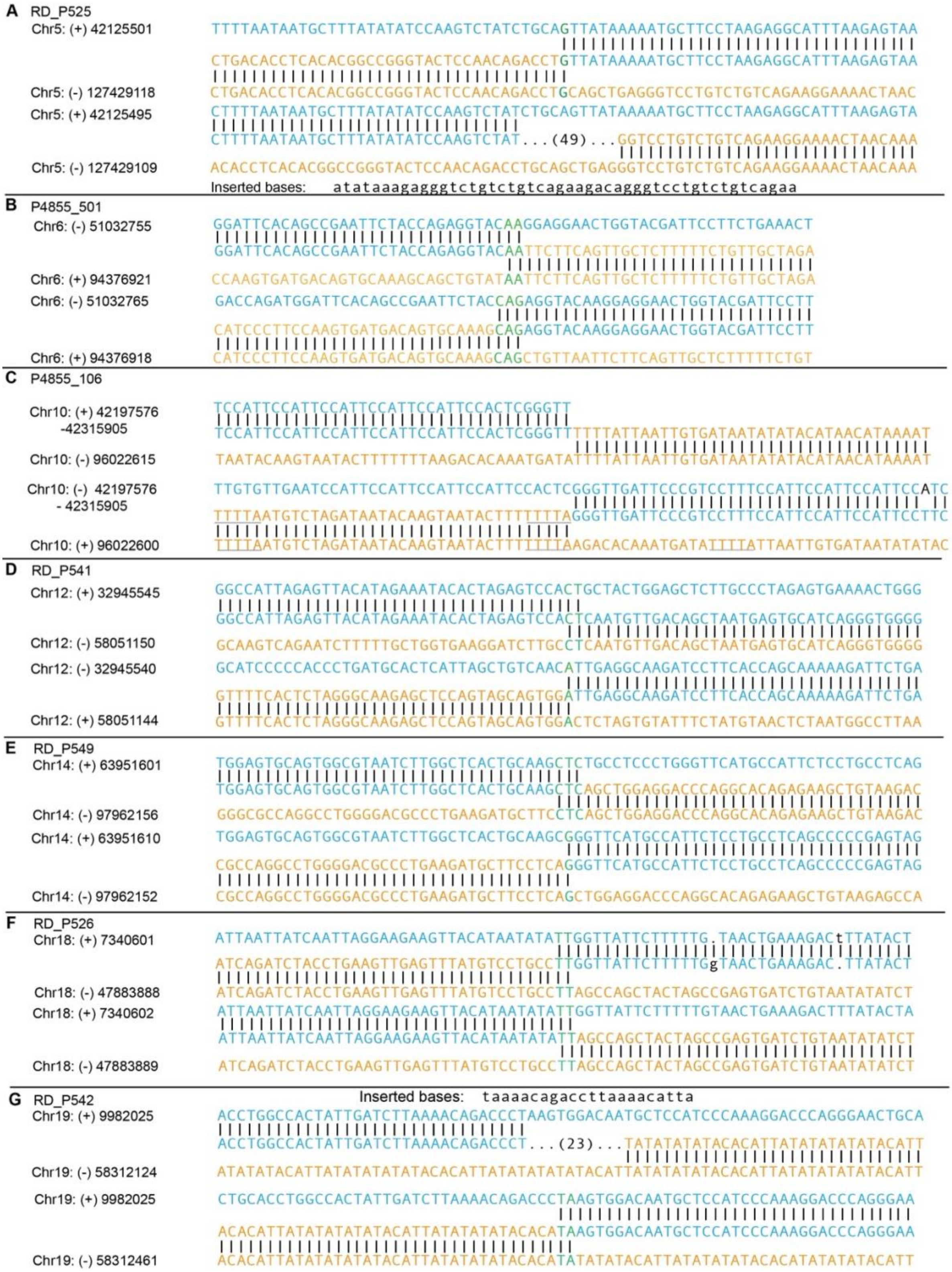

Supplemental Fig. S2: Breakpoint sequences of inversions. **A)** Inversion 5 (RD_P525), **B)** Inversion 6 (P4855_501), **C)** Inversion 10 (P4855_106), where the one of the breakpoint sequences at 10:42197576-42315905 could not be fully pinpointed, **D)** inversion 12 (RD_P541), **E)** inversion 14 (RD_P549), **F)** inversion 18 (RD_P526) and **G)** inversion 19 (RD_P542) breakpoint sequences. Microhomology is indicated in green, unmatching

sequences in black and repeat sequences is underlined. The direction and positions are indicated at the left side of the figure.

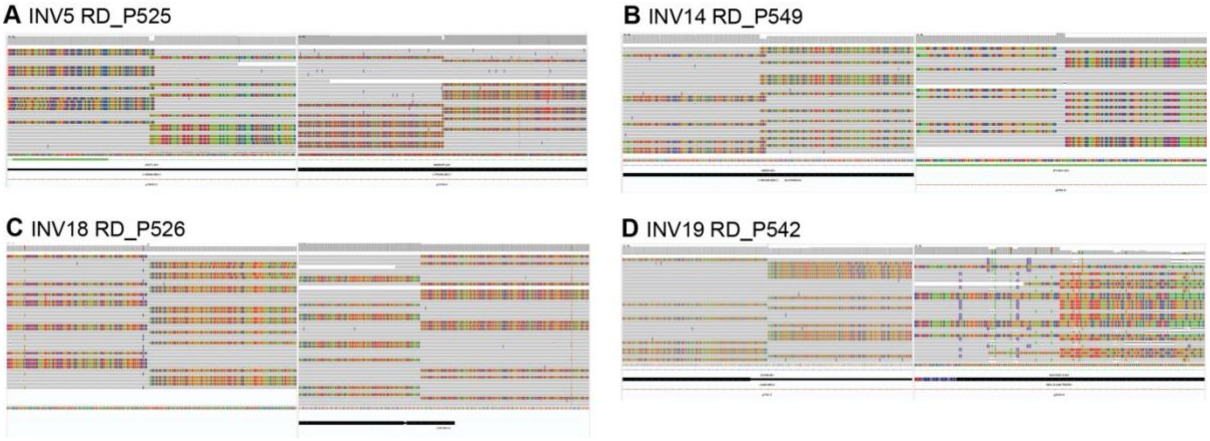

Supplemental Fig. S3: IGV images of resolved inversions (INV) with breakpoints in mappable regions **A)** INV5 (RD_P525), **B)** INV14 (RD_P549), **C)** INV18 (RD_P526) and **D)** INV19 (RD_P542).

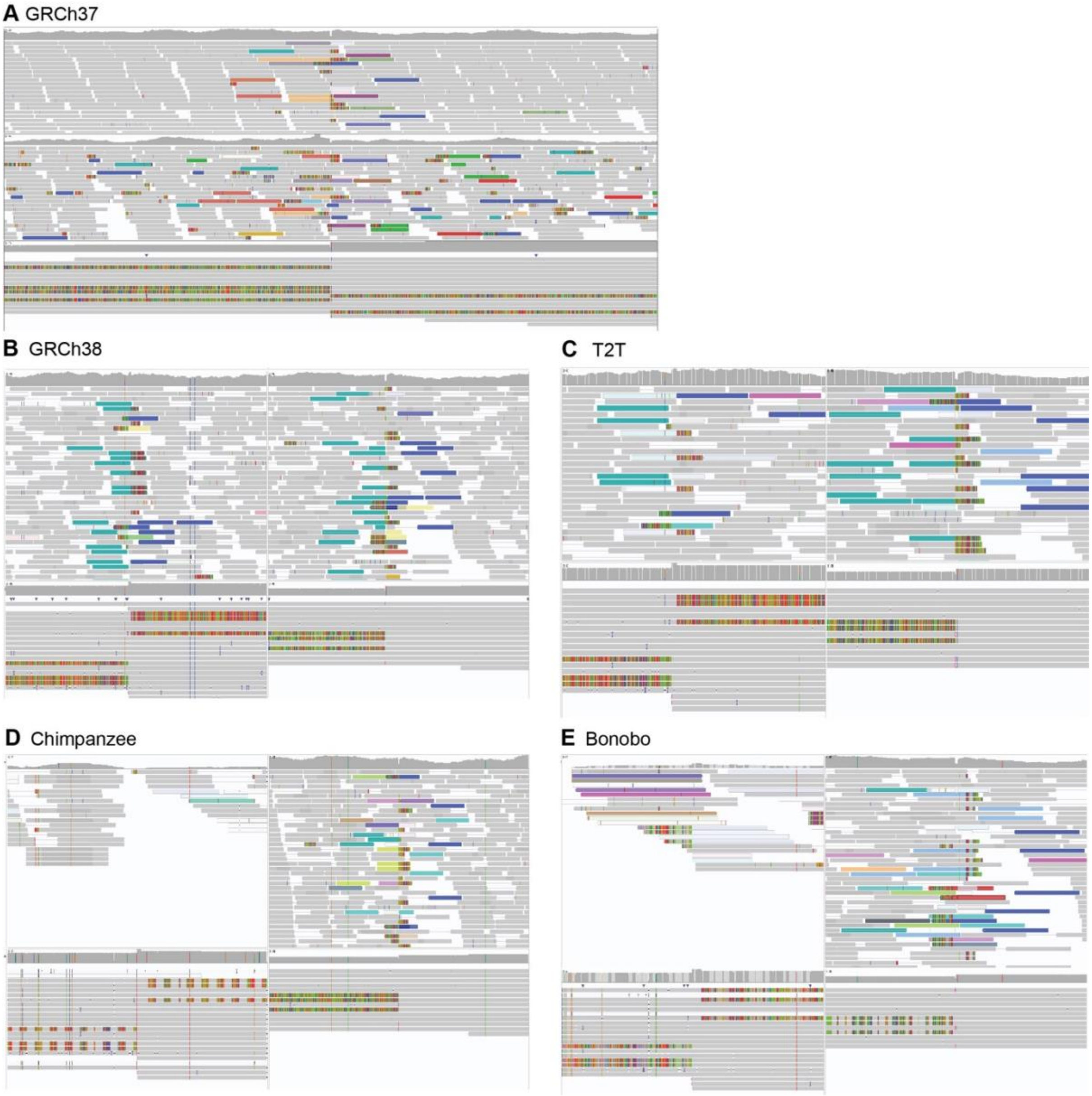

Supplemental Fig. S4: Integrated genomics viewer (IGV) images of the inversion 6 breakpoint regions in short-read (upper), linked-read (middle, only A) and long-read (lower) data in GRCh37 **(A)**, GRCh38 **(B)**, T2T **(C)**, chimpanzee **(D)** and bonobo **(E)**.

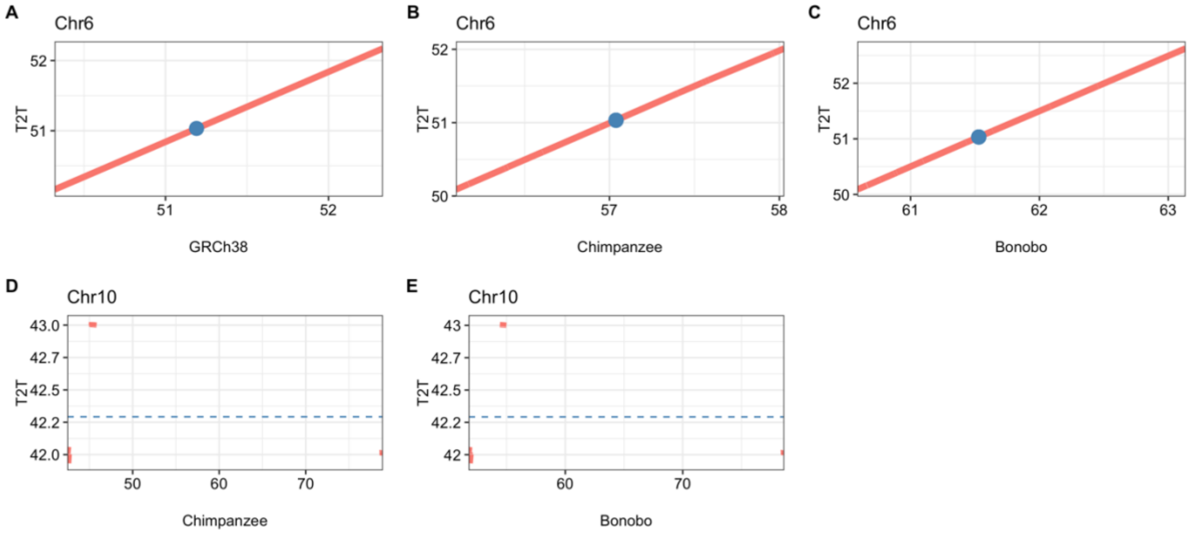

Supplemental Fig. S5: Comparison of the inversion breakpoint region on chromosome 6p and chromosome 10q across reference genomes. The reference sequence on chromosome 6 affected by an inversion compared in **A)** GRCh38-T2T, **B)** Chimpanzee to T2T and **C)** Bonobo to T2T. The reference sequence on chromosome 10 affected by an inversion compared in **D)** Chimpanzee to T2T and **E)** Bonobo to T2T.

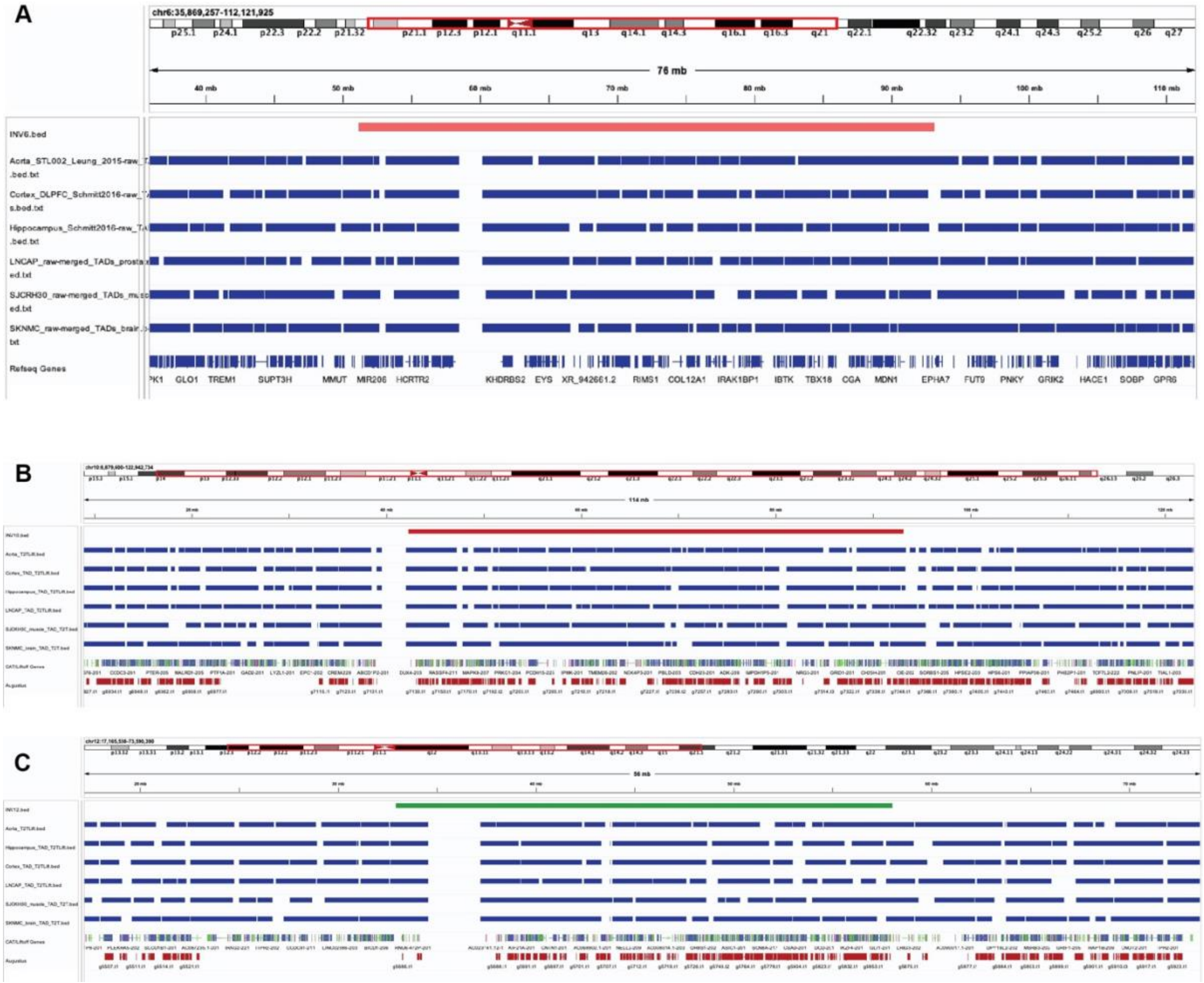

Supplemental Fig. S6: Topologically Associated Domains and A) Inversion 6, B) Inversion 10 and C) Inversion 12.

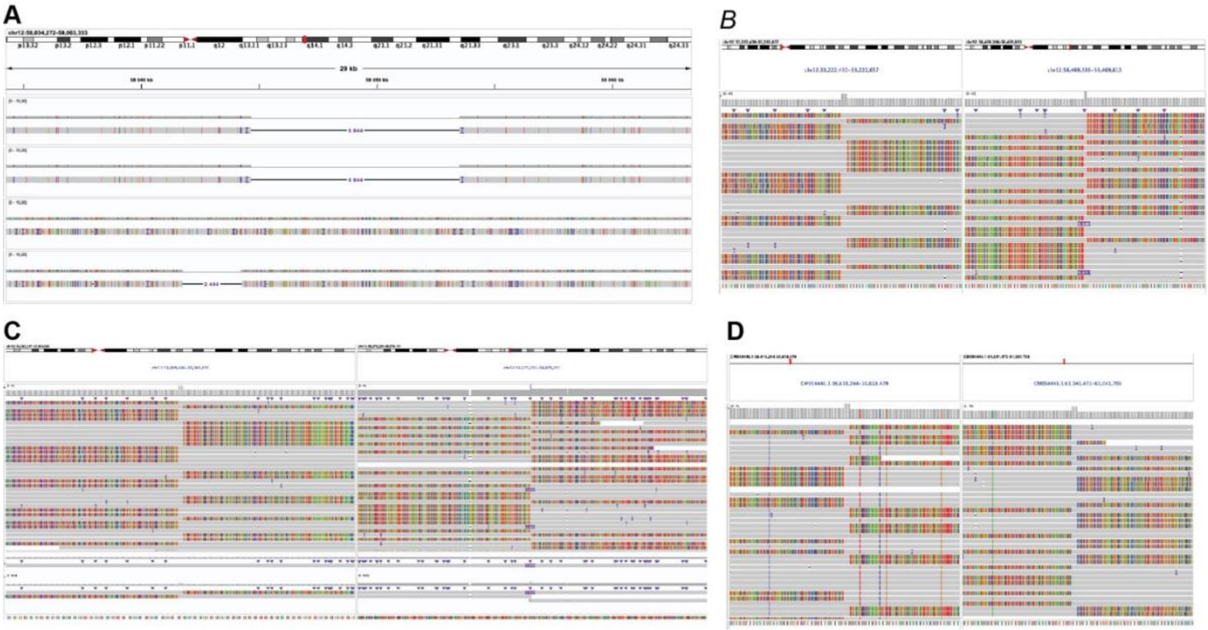

Supplemental Fig. S7: Inversion affecting chromosome 12 in case RD_P541. A) The inversion breakpoint region on 12q in GRCh38, GRCh37, chimpanzee and bonobo aligned to T2T. **B)** Breakpoint regions in GRCh37. **C)** Breakpoint regions in GRCh38. **D)** Breakpoint regions in chimpanzee.

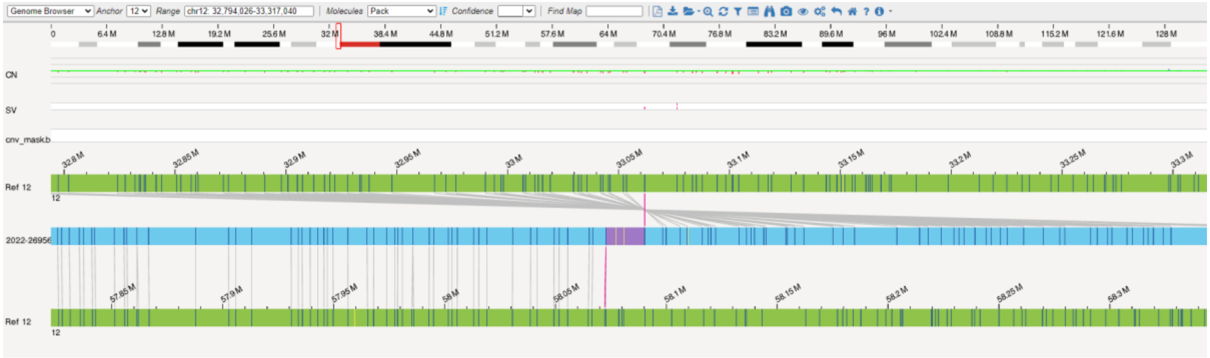

Supplemental Fig. S8: Optical genome mapping of chromosome 12, RD_P541.

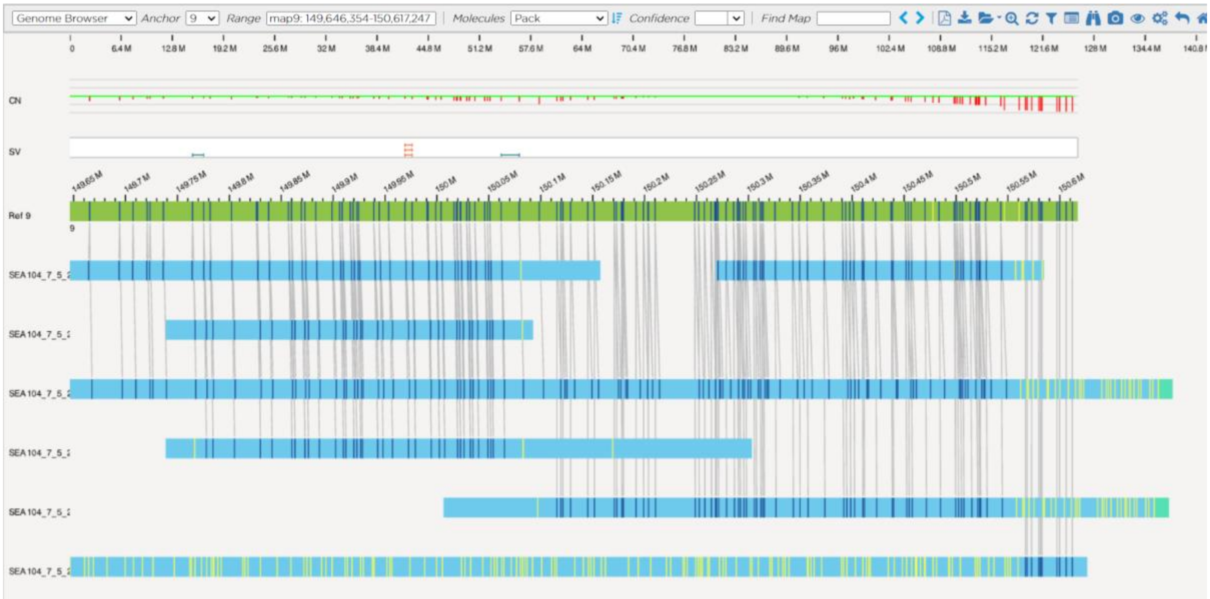

Supplemental Fig. S9: Optical Genome mapping of the chromosome 9, BH16643-1. Pinpoints of the inversion can be observed as unaligned regions.

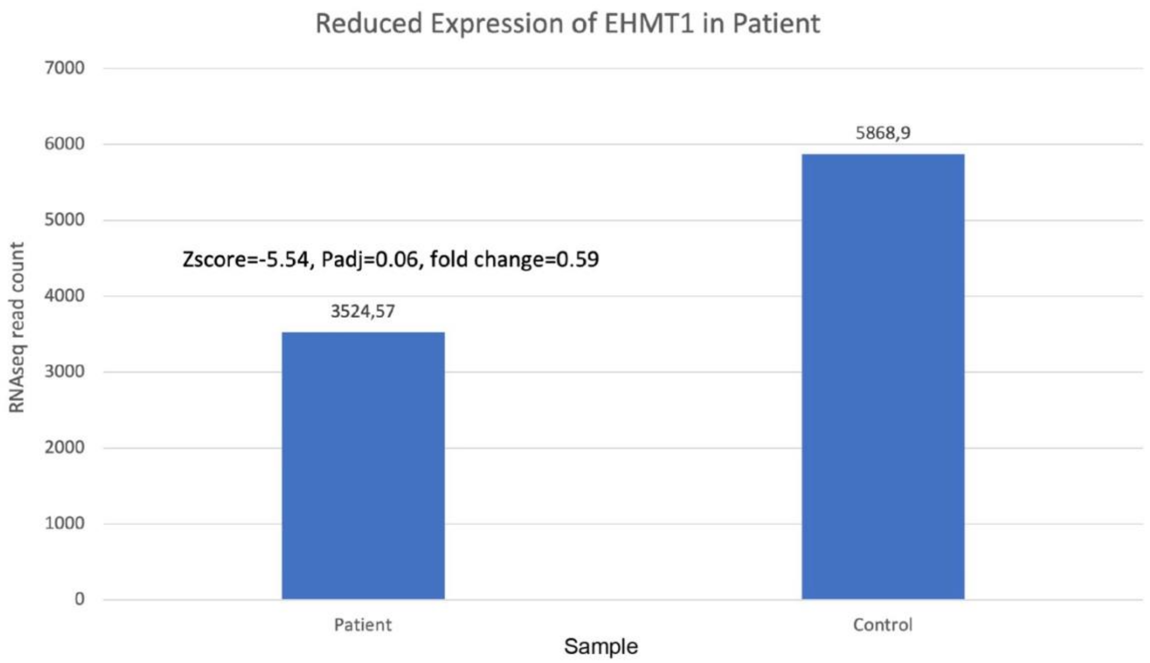

Supplemental Fig. S10: Reduced expression of EHMT1 in BH16643-1.

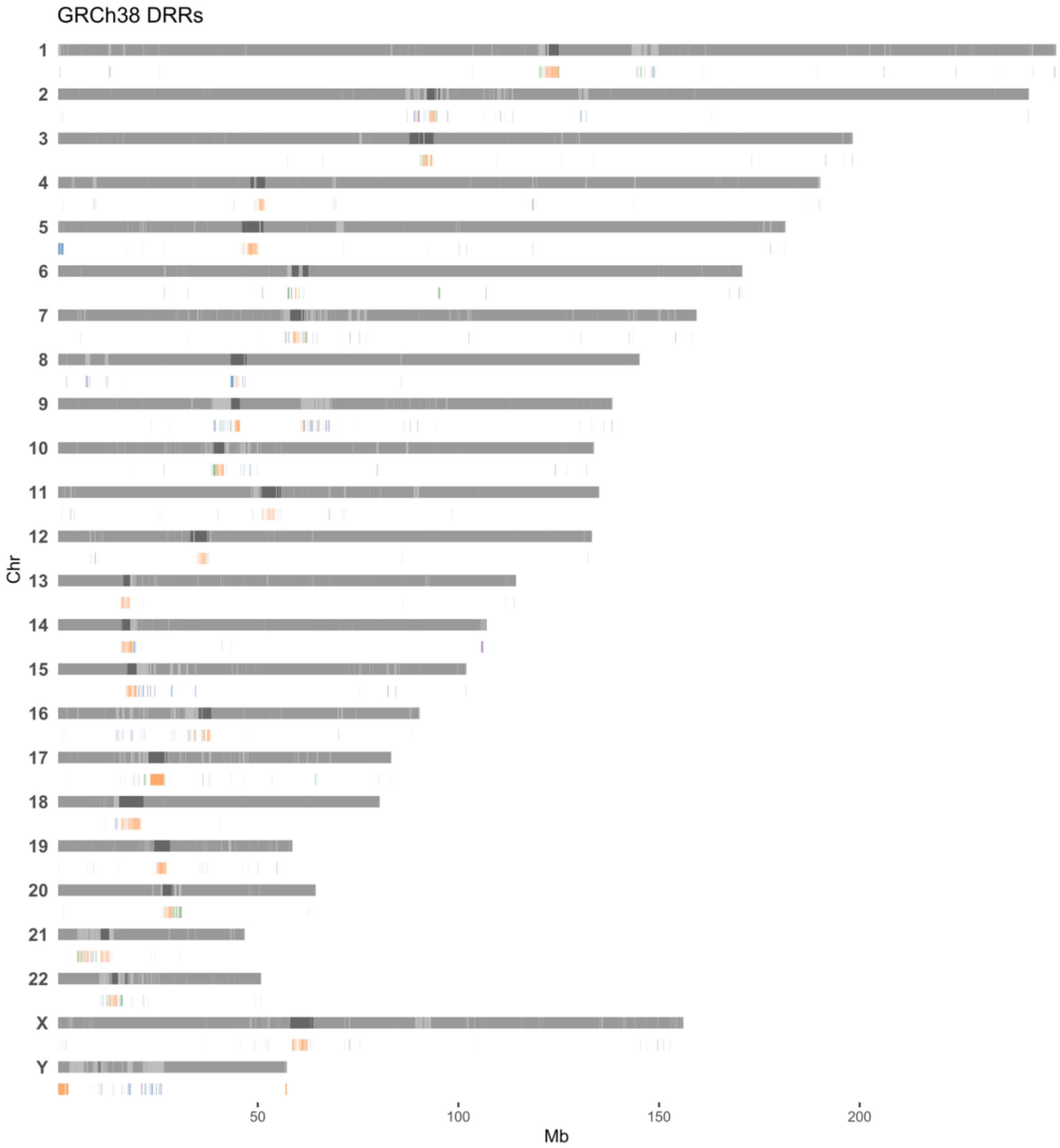

Supplemental Fig. S11: DRR analysis. Bar plot of all DRRs in GRCh38. Orange indicating all compared genomes, blue bonobo, purple chimpanzee, green GRCh37 and brown T2T.

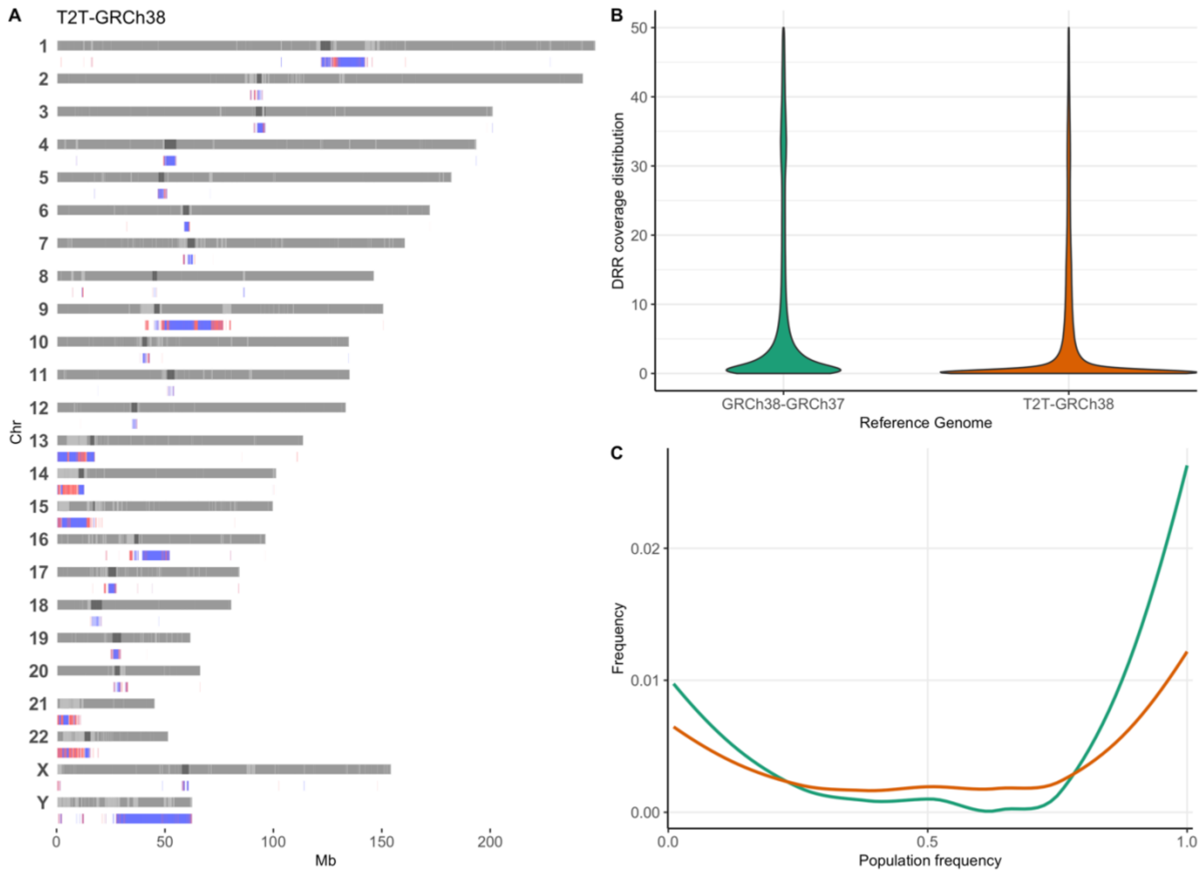

Supplemental Fig. S12: Distribution of DRRs in 100 Swedish individuals. A) DRRs between T2T-GRCh38 and their presence in Swedish individuals. Blue indicating absent (<8X) and red present (>8X and <100X). **B)** Violin plot of coverage across the respective DRRs in 100 Swedish individuals. **C)** The distribution of population frequencies of the detected GRCh38-GRCh37 (green) and T2T-GRCh38 (orange) DRRs.

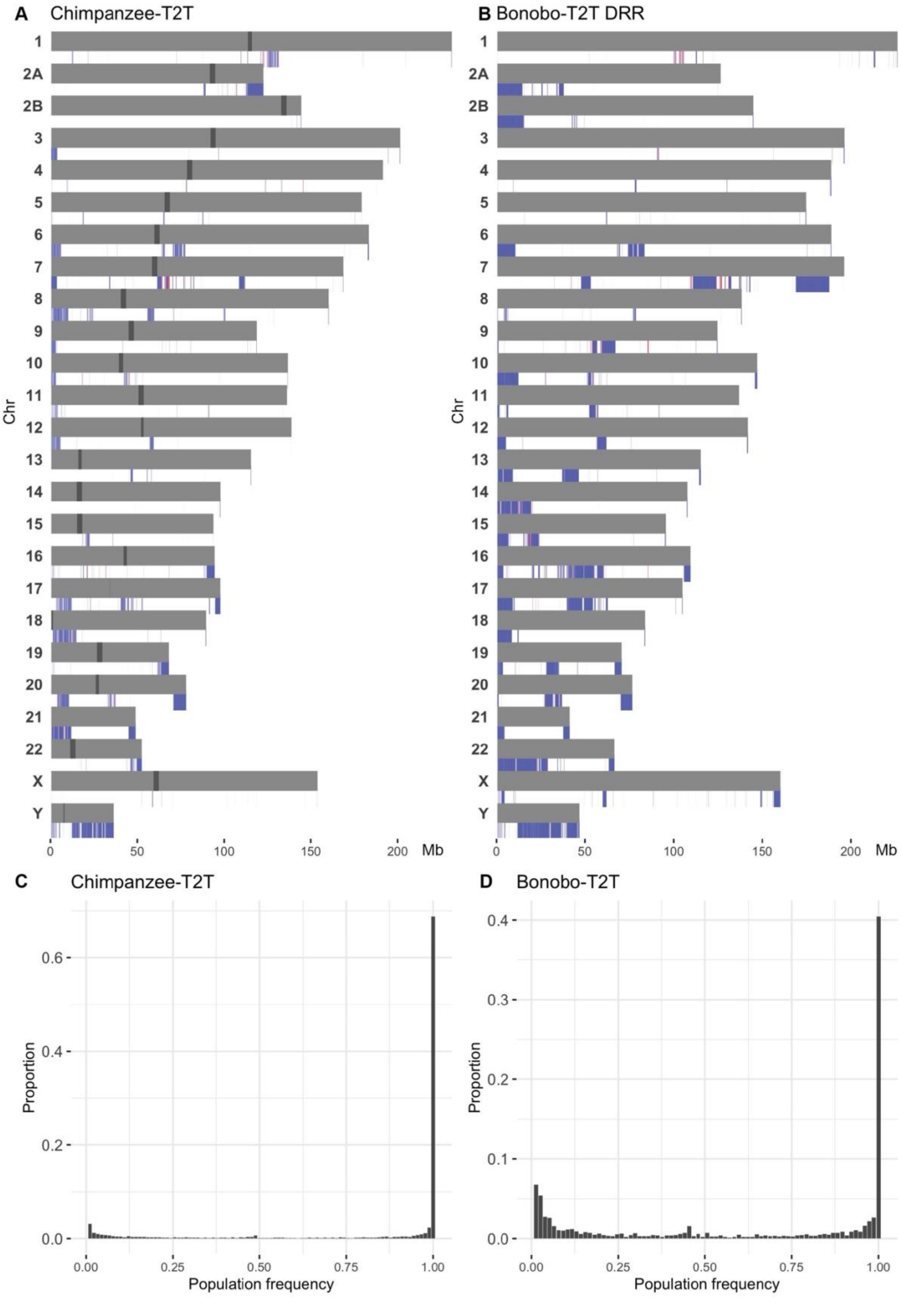
A Chimpanzee-T2T
B Bonobo-T2T DRR
Chr
1
2A
2B
3
4
5
6
7
8
9
10
11
12
13
14
15
16
17
18
19
20
21
22
X
Y
0
50
100
150
200
Mb
C Chimpanzee-T2T
D Bonobo-T2T
Proportion
Population frequency
0.00
0.25
0.50
0.75
1.00
0.0
0.2
0.4
0.6
0.1
0.3

Supplemental Fig. S13: Bar plots A) DRRs in Chimpanzee-T2T **B)** DRRs between Bonobo-T2T. Blue indicating absent in Swedish individuals and red indicate present. **C)** Population frequency of DRRs of Chimp-T2T **D)** DRRs between Bonobo-T2T.

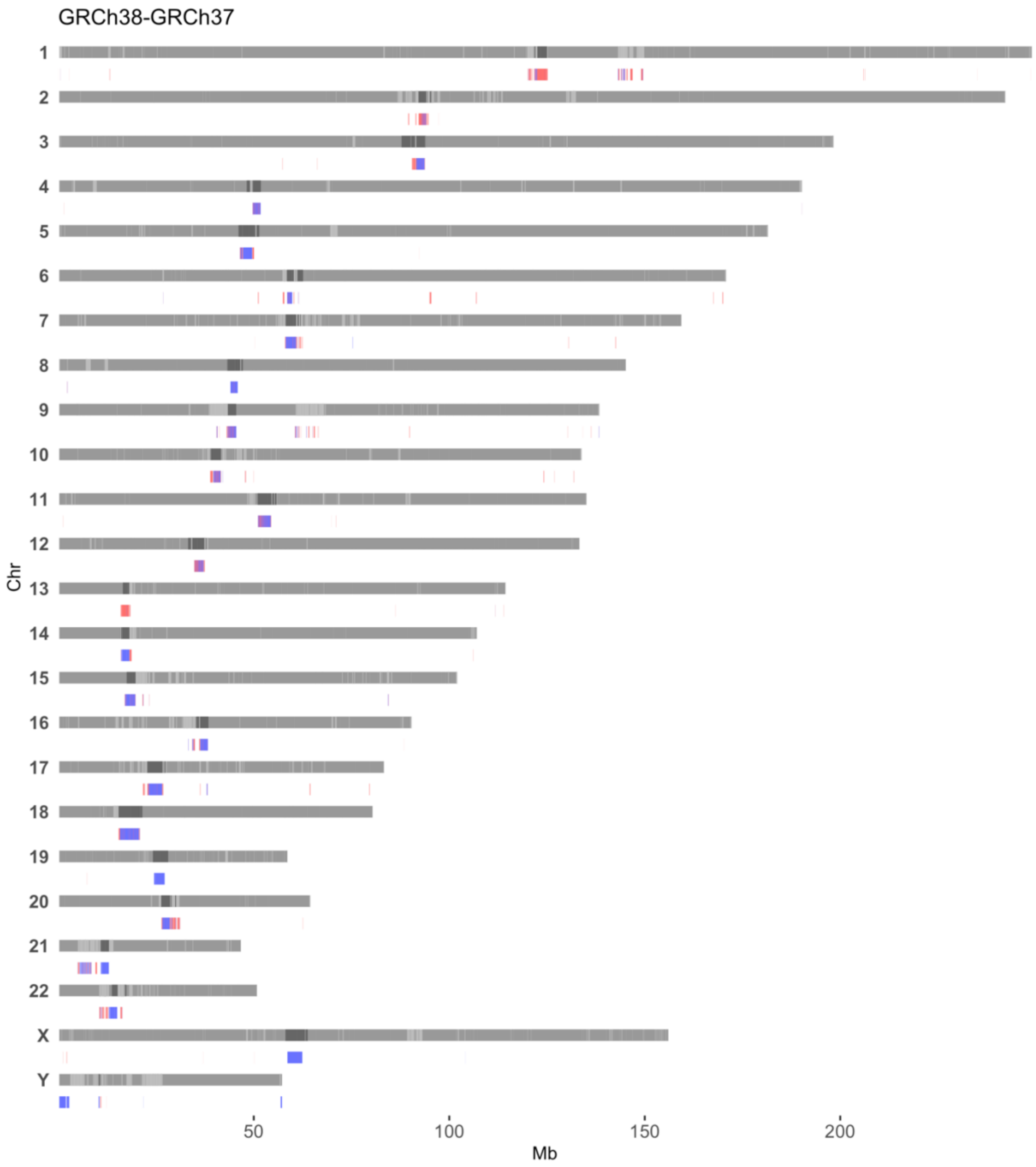

Supplemental Fig. S14: DRRs of GRCh38-GRCh37 and their presence in Swedish individuals. Blue indicating absent and red present.

Supplemental Table S2: Median, minimum and maximum length of DRRs (Mbp).

		Median	Min	Max

Template	**GRCh38**	20	10	2270
	**T2T**	20	10	930
	**Chimpanzee**	10	10	450
	**Bonobo**	10	10	300

Supplemental Table S3: Coverage thresholds determining genomics copies.

Coverage	Copies
8-24x	1
25-38	2
39-45	3
46-100	>3

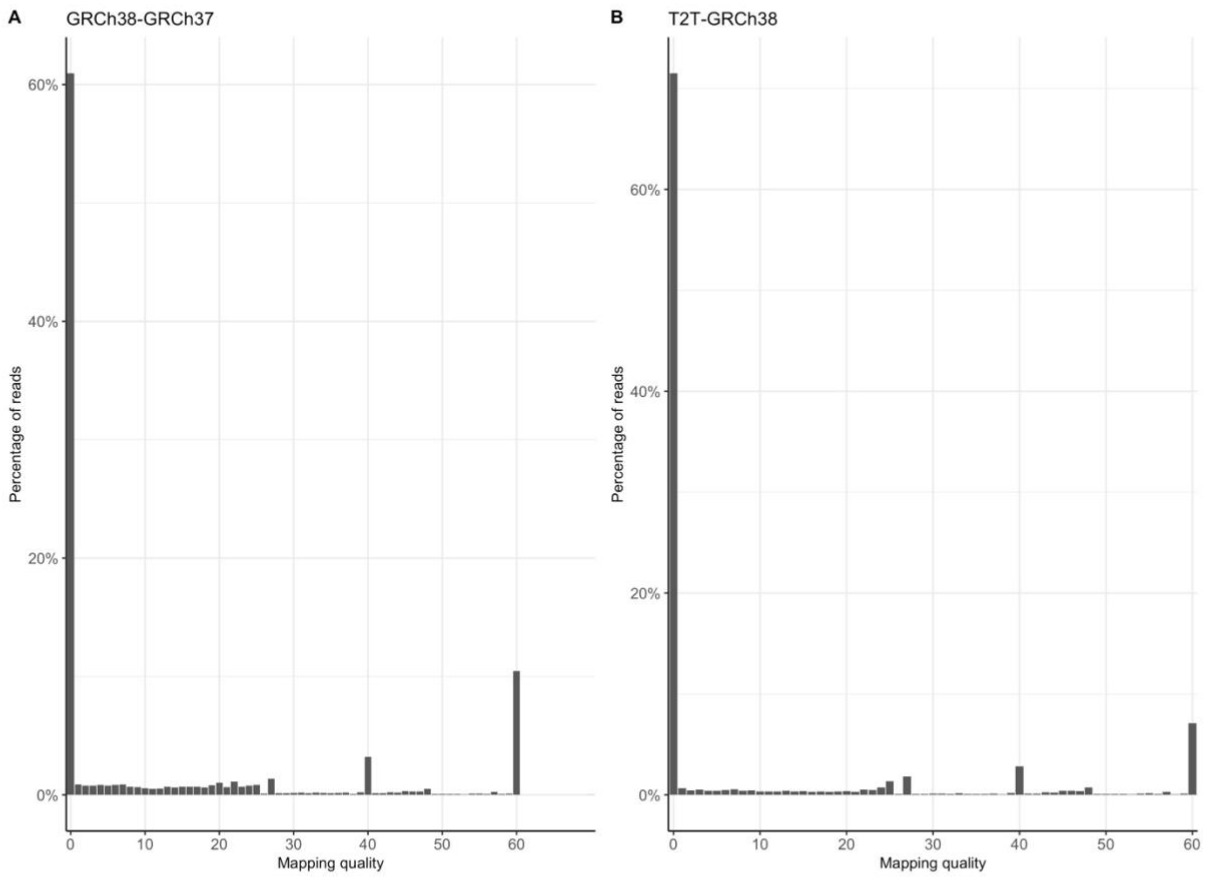

Supplemental Fig. S15: Distribution of mapping quality of reads aligned to DRRs in A) GRCh38-GRCh37 and **B)** T2T-GRCh38.

Supplemental Document 1: Detailed clinical description of BH16643-1

BH16643-1 is a 4-year-old girl with hypotonia and global developmental delay. Pregnancy was complicated by polyhydramnios. She was born at 39 weeks gestational age (birth weight 3317 grams, birth length 49.5 cm, and FOC 33 cm). She required phototherapy for hyperbilirubinemia and otherwise received routine care in the nursery. As to her development, she gained head control at 7 months, rolled over at 9 months, sat up unsupported at 12 months, and learned to sit up independently when she was 2.5 years old. At age 4 years, she can crawl and bear weight on feet with support, but she is not cruising or walking. She reached to grab an object at age 7 months and developed pincer grasp at age 16 months. She is non-verbal. She shows self-harmful behaviors when frustrated or irritated. There is no history of developmental regression or seizures. The medical history is otherwise significant for feeding difficulties, initially presenting with difficulty latching to breast and poor suck, followed by dysphagia and persistent gastroesophageal reflux. She has chronic constipation. She has a congenital heart defect, including a small atrial septal defect that closed, and mild pulmonary valve stenosis. She has bilateral optic nerve atrophy and intermittent exotropia, requiring corrective lenses. Her history is also notable for recurrent respiratory infections, associated with frequent nasal congestion and ear infections requiring repeated pressure equalizing tube placement. Family ethnicity is mixed Caucasian, Native American and Cajun, and the family history was non-contributory. On physical examination, her growth was within the normal limits, and she was noted to have dysmorphic craniofacial features, including midface retrusion, everted lower lip and prognathism. The neurological exam demonstrated hypotonia and normal muscle strength. The patient had an abnormal brain magnetic resonance imaging (MRI) at age 2 years that showed hypoplastic corpus callosum and pontocerebellar hypoplasia. Spinal MRI revealed closed sacral dysraphism (spina bifida occulta) at S2-S3 that was felt to be an incidental finding. Electroencephalography (EEG) was normal. Metabolic labs, including ammonia, lactate, plasma amino acids, urine organic acids, acylcarnitine analysis, urine amino acids, very long chain fatty acids, 7-dehydrocholesterol, and congenital disorders of glycosylation biochemical screening panel did not show significant abnormalities. Genetic testing included a chromosomal microarray analysis (CMA), trio exome sequencing and mitochondrial DNA sequencing, Prader Willi methylation study, *SMN1/SMN2* dosage analysis and *DMPK* repeat expansion analysis, all with normal results. Through clinical genome sequencing conducted in the UDN the patient was found to be a carrier for Friedreich ataxia, with a heterozygous *FXN* repeat expansion (approximately 1050 repeats) that was inherited from her mother. Chromosome analysis in blood and skin fibroblasts identified a heterozygous, paracentric inversion on the long arm of chromosome 9 [46,XX,inv(9)(q12q34.3)]. Parental chromosome analysis was normal, suggesting the inversion arose *de novo* in the patient.

Supplemental Code:

## Align sr ##

```
bwa mem -p -t 16 <ref> <fastq>

# Align and SV calling of linked-read
longranger wgs –id <id> --reference <ref> --fastq <fastq> --vcmode freebayes
```

## Denovo analysis ##

```
# YAK
yak count -k31 -b37 -t16 -o $2.yak <fasta>

# Hifiasm
hifiasm -o $2 -t 16 $fastq

# GFA to FA
awk '/^S/{print ">"$2;print $3}' hap.gfa > $2.fa

# align FA
minimap2 -R "@RG\tID:$2\tSM:$2" -a -t 16 --MD -x map-pb -asm5 -Y -y $ref $2.fa |
samtools view -Sbh - | samtools sort -m 4G -@16 - > $2.bam
samtools index $2.bam

# SVIM
svim-asm diploid $PWD <hap1> <hap2> $ref --sample $3

# Quast
python quast <hap1> <hap2>  -o $3.quast -t 16 -r $ref
```

## Reference genome anlaysis ##

```
minimap2 -cx asm5 template.fa query.fa > aln.paf
```

## Find DRRs ##

Create BAM file ref-to-ref /ref1=ref ref2=asm

```
minimap2 -ax asm5 <ref1> <ref2> | samtools view -Sbh - > tmp.ref1.ref2
samtools sort -m 4G -@1 tmp.ref1.ref2 > ref1_ref2.bam
samtools index ref1_ref2.bam
```

Run TIDDIT coverage module to find gaps

```
tiddit=container.sif
singularity exec --bind /dataset $tiddit tiddit --cov --bam ref1_ref2.bam -
o  ref1_ref2.cov -z 10000
```

Remove known gap regions in template

```
bedtools intersect -v -a ref1_ref2.cov -b gapsRef1.bed  > ref1_ref2.novelgap.bed
```

Collect all gap regions : coverage of 0 across the bin

```
grep -P '\t0\.0' ref1_ref2.novelgap.bed >> ref1_ref2.novelgap.bed
```

## Count DRRs

```
import sys
import statistics

"""
find Mbp of DRR sequence that have low-medium-high coverage
AND
find amount average coverage across gap regions
AND
```

```
identify variable regions

INPUT: cov file from TIDDIT
"""

mychr = list(range(0,23))
mychr = [str(i) for i in mychr]
mychr.append('X')
mychr.append('Y')

genome='T2T'
#genome ='GRCh38'
#genome='GRCh37'
#genome='Chimpanzee'
#genome='Bonobo'

def collect_cov(file): # collects info from file

	covdict = {}
	bindict = {}
	for line in open(file):
		tabline = line.rstrip('\n').split()
		chr=tabline[0]

		#if chr not in mychr:
		#	continue

		start = tabline[1]
		end = tabline[2]
		cov = float(tabline[3])

		if chr not in covdict:
			covdict[chr] = {}
			bindict[chr]={}
		if start not in covdict[chr]:
			covdict[chr][start] = []
			bindict[chr][start] = end

		covdict[chr][start].append(cov)
		lst=[chr, start, end, str(cov)]
		#print('\t'.join(lst))
	return covdict, bindict


def count_presence(covdict, bindict):

	for chr in covdict:
		for s in covdict[chr]:
```

```
                average = sum(covdict[chr][s])/len(covdict[chr][s])
                covlist = covdict[chr][s]

                #count presence
                lowcov = len([i for i in covlist if i <= 8])
                highcov = len([i for i in covlist if i >8 and i <100 ])

                if highcov >= 5:
                        status = 'Common'

                else:
                        status = 'Absent'


                lst = [chr, s, bindict[chr][s], str(average), genome, status]
                print('\t'.join(lst))
        return

def find_amount(file):
        amount=[]
        for line in open(file):
                am = int(line.rstrip('\n'))
                amount.append(am)
        mean = sum(amount)/len(amount)
        median = statistics.median(amount)

        meanMBP=mean*10000/1000000
        medianMBP=median*10000/1000000

        print('median', medianMBP)
        print('mean', meanMBP)

find_amount(sys.argv[1]) #find amount of DRRs
all_cov= collect_cov(sys.argv[1]) #coverage across all regions
count_presence(all_cov[0], all_cov[1])
```

Multimapping reads

```
import sys
import pysam

reference_filename='/sw/data/uppnex/ToolBox/hg38bundle/Homo_sapiens_assembly38.fast
a' )
bamfile = pysam.AlignmentFile(sys.argv[2], "rc", reference_filename='reference.fna')
regions=sys.argv[1]

def myregions(file):
        reg= {}
```

```
	for line in open(file):
		chr=line.split(':')[0]
		if chr == 'chrMT':
			continue
		start=int(line.split(':')[-1].split('-')[0])
		end=int(line.rstrip('\n').split(':')[-1].split('-')[1])
		if chr not in reg:
			reg[chr]={}

		if start not in reg[chr]:
			reg[chr][start]=end

	return reg




def findMM(bam, regdict):
	mm={}
	flagmm={}
	myqual=[256,2048,2057, 2056, 2129, 2209, 2193]
	for chr in regdict:
		for start in regdict[chr]:
			end=regdict[chr][start]
			for read in bamfile.fetch(chr, start, end, until_eof=True):
				flag= int(read.flag)
				#print(flag)
				name=read.query_name
				if flag in myqual:
					if name not in flagmm:
						flagmm[name]=0
					flagmm[name]+=1
				if name not in mm:
					mm[name]=0
				mm[name]+=1
	return mm, flagmm


regionsdict=myregions(regions)
multimapping= findMM(bamfile, regionsdict)

total=0
for r in multimapping[1]:
	if multimapping[1][r] > 1:
		total += 1

perc1=len(multimapping[1])/len(multimapping[0])*100
print(str(bamfile), len(multimapping[0]), len(multimapping[1]), perc1, total )
```

Mapping quality

```
import sys
import pysam

bamfile = pysam.AlignmentFile(sys.argv[2], "rc",
reference_filename='/sw/data/uppnex/ToolBox/hg38bundle/Homo_sapiens_assembly38.fast
a' )
#bamfile = pysam.AlignmentFile(sys.argv[2], "rc",
reference_filename='/proj/sens2017106/nobackup/kristine/reference_stuff/GCF_009914755.
1_T2T-CHM13v2.0_genomic.fna')
regions=sys.argv[1]

def myregions(file):
	reg= {}
	for line in open(file):
		chr=line.split(':')[0]
		if chr == 'chrMT':
			continue
		start=int(line.split(':')[-1].split('-')[0])
		end=int(line.rstrip('\n').split(':')[-1].split('-')[1])
		if chr not in reg:
			reg[chr]={}

		if start not in reg[chr]:
			reg[chr][start]=end

	return reg

def findMQ(bam, regdict):
	mq=[]
	for chr in regdict:
		for start in regdict[chr]:
			end=regdict[chr][start]
			for read in bamfile.fetch(chr, start, end, until_eof=True):
				mapq= str(read.mapping_quality)
				mq.append(mapq)
				print('\t'.join([str(chr), str(start), str(end), str(mapq)]))
	return mq


regionsdict=myregions(regions)
mappingquality= findMQ(bamfile, regionsdict)
```
